# Transcriptomic Bioinformatic Analyses of Atria Uncover Involvement of Pathways Related to Strain and Post-translational Modification of Collagen in Increased Atrial Fibrillation Vulnerability in Intensely Exercised Mice

**DOI:** 10.3389/fphys.2020.605671

**Published:** 2020-12-23

**Authors:** Yena Oh, Sibao Yang, Xueyan Liu, Sayantan Jana, Farzad Izaddoustdar, Xiaodong Gao, Ryan Debi, Dae-Kyum Kim, Kyoung-Han Kim, Ping Yang, Zamaneh Kassiri, Robert Lakin, Peter H. Backx

**Affiliations:** ^1^Department of Biology, York University, Toronto, ON, Canada; ^2^Department of Physiology, University of Toronto, Toronto, ON, Canada; ^3^Department of Cellular and Molecular Medicine, Faculty of Medicine, University of Ottawa, Ottawa, ON, Canada; ^4^University of Ottawa Heart Institute, Ottawa, ON, Canada; ^5^Department of Cardiology, China-Japan Union Hospital of Jilin University, Changchun, China; ^6^Department of Physiology, Cardiovascular Research Center, University of Alberta, Edmonton, AB, Canada; ^7^Donnelly Centre, University of Toronto, Toronto, ON, Canada; ^8^Lunenfeld-Tanenbaum Research Institute, Sinai Health System, Toronto, ON, Canada

**Keywords:** atrial fibrillation (AF), RNA sequencing (RNA-seq), tumor necrosis factor, inflammation, collagen, mechanotransduction, heart, exercise

## Abstract

Atrial Fibrillation (AF) is the most common supraventricular tachyarrhythmia that is typically associated with cardiovascular disease (CVD) and poor cardiovascular health. Paradoxically, endurance athletes are also at risk for AF. While it is well-established that persistent AF is associated with atrial fibrosis, hypertrophy and inflammation, intensely exercised mice showed similar adverse atrial changes and increased AF vulnerability, which required tumor necrosis factor (TNF) signaling, even though ventricular structure and function improved. To identify some of the molecular factors underlying the chamber-specific and TNF-dependent atrial changes induced by exercise, we performed transcriptome analyses of hearts from wild-type and TNF-knockout mice following exercise for 2 days, 2 or 6 weeks of exercise. Consistent with the central role of atrial stretch arising from elevated venous pressure in AF promotion, all 3 time points were associated with differential regulation of genes in atria linked to mechanosensing (focal adhesion kinase, integrins and cell-cell communications), extracellular matrix (ECM) and TNF pathways, with TNF appearing to play a permissive, rather than causal, role in gene changes. Importantly, mechanosensing/ECM genes were only enriched, along with tubulin- and hypertrophy-related genes after 2 days of exercise while being downregulated at 2 and 6 weeks, suggesting that early reactive strain-dependent remodeling with exercise yields to compensatory adjustments. Moreover, at the later time points, there was also downregulation of both collagen genes and genes involved in collagen turnover, a pattern mirroring aging-related fibrosis. By comparison, twofold fewer genes were differentially regulated in ventricles vs. atria, independently of TNF. Our findings reveal that exercise promotes TNF-dependent atrial transcriptome remodeling of ECM/mechanosensing pathways, consistent with increased preload and atrial stretch seen with exercise. We propose that similar preload-dependent mechanisms are responsible for atrial changes and AF in both CVD patients and athletes.

## Introduction

Atrial fibrillation (AF) is the most common supraventricular tachyarrhythmia seen in clinical practice (Calkins et al., 2018), with its prevalence predicted to double by 2060 (Krijthe et al., 2013). AF is easily identified in electrocardiographic (ECG) recordings by the presence of rapid rates (typically > 110 beats/min) coupled with regular-irregular QRS complexes and is associated with impaired cardiac output regulation, non-pumping “quivering” atria, and an increased risk of stroke. The etiology of AF is complex, with most patients being elderly and also suffering from cardiovascular diseases (esp., hypertension, heart failure, valve disease) or having increased risk for cardiovascular disease (sleep apnea, hyperthyroidism, obesity, and diabetes) (Odutayo et al., 2016; Staerk et al., 2017). A common physiological feature of all these conditions, including aging, is increased filling pressures (De Jong et al., 2011; Park et al., 2014) which is believed to drive the atrial fibrosis, inflammation and hypertrophy invariably seen in AF patients. The importance of addressing the AF epidemic is highlighted by the > twofold increase in all-cause mortality seen in patients with AF.

Paradoxically, the risk of AF is also increased in veteran endurance athletes (Mont et al., 2002; Redpath and Backx, 2015), despite well-established evidence of beneficial physiological remodeling of the ventricles. Although the underlying basis for exercise-induced AF and its differential effects on the atria and ventricles are unclear, it is well known that intense exercise is associated with marked elevations in filling pressure (Reeves et al., 1990). Moreover, rodent models have established that endurance exercise causes atrial fibrosis, inflammation, and hypertrophy (Guasch et al., 2013; Aschar-Sobbi et al., 2015), as seen in persistent AF patients (Oakes et al., 2009; Qu et al., 2009; Gramley et al., 2010). We previously demonstrated that the exercise-induced atrial changes were prevented by pharmacological and genetic blockade of tumor necrosis factor (TNF), a mechanosensitive and pro-inflammatory cytokine (Aschar-Sobbi et al., 2015; Lakin et al., 2019). However, when TNF blockade was introduced 3 weeks after the beginning of intense exercise, cardioprotection was lost, suggesting that pathways linked to exercise-induced adverse atrial remodeling occur early in the response to exercise.

In this study, we performed bioinformatic analyses of transcriptomic changes in atria and ventricles induced by endurance exercise in wild-type and TNF knockout mice. Our results demonstrate that exercise induces TNF-dependent differential activation (enrichment) of pathways associated with mechanosensitive ECM remodeling that are time-dependent and differ between atria and ventricles, in a manner consistent with preferential stretch of atria in response to exercise-induced elevations in venous pressure. Our findings provide insight into the chamber-specific roles of TNF and mechanical strain in cardiac changes induced by exercise and support the general conclusion that exercise-induced adverse atrial remodeling is preload-dependent as seen in AF associated with aging and poor cardiovascular health.

## Materials and Methods

### Experimental Animals and Endurance Exercise Training Models

This study was carried out in accordance with the recommendations of the Canadian Council of Animal Care. The protocol was approved by the Division of Comparative Medicine at the University of Toronto and York University Animal Care Committee. Mice swam for 2 days (4-sessions), 2 or 6 weeks against water currents in containers, as described previously (Aschar-Sobbi et al., 2015). For the 6 week group, 6 week old CD1 male mice (body weight = 28–34 g, Charles River Laboratories) were acclimatized by swimming twice daily for 30 min (separated by 4 h) after which the duration of the swims was increased by 10 min per day until the duration reach 90 min per swim. Thereafter, the mice swam 2 times per day for 90 min/session for 6 weeks. The swim protocol was similar for the 2 week group, except mice only swam for 2 weeks after acclimatization. These 2 week mice were bred in-house after backcrossing TNF knockout (TNF-KO) mice (c57b, Taconic model #1921) into a CD1 background a minimum of 8 times. After reaching 10 weeks of age, wild-type (WT) mice and their TNF-KO littermates were acclimatized as described for the 6 week mice and swam for 2 weeks. The breeding and housing for the 2 day group were as described for the 2 week group. At 10–12 weeks of age, these mice were familiarized for 3 days with a 10 min swim per day, followed by two consecutive days of twice daily, 90 min swims. The sedentary mice for all groups consisted of the age-matched animals who were placed in swim containers without a water current for 5 min each session to ensure similar handling.

### Tissue Harvesting

Prior to harvesting of atrial and ventricular tissue, 0.2 ml of heparin was injected intraperitoneally to prevent blood clotting. After 5 min, mice were anesthetized using 2.5% isoflurane and sacrificed via cervical dislocation. Hearts were quickly excised and placed into cold phosphate-buffered saline (PBS) to prevent protein or RNA degradation as well as cell apoptosis. In cold PBS, the left atrial appendage (LAA) and left ventricular (LV) free wall were separated and collected for RNA extraction. For our 2 day swim protocol, tissue was harvested 2 h after last swim. For 2 and 6 week studies, tissue was harvested 24 h after the last swim session.

### RNA Extraction

Total RNA was extracted for both atria and ventricles using RNeasy Mini Kit (Qiagen), where the silica membrane used for this protocol removes RNA shorter than 200 nucleotides, including 5S/5.8S rRNAs, microRNAs and all tRNAs. All RNA samples were stored at −80°C until use. Initial RNA quantity and quality analyses were done using the Nanodrop2000 spectrophotometer (Thermo Fisher Scientific). The integrity and concentration of the RNA was determined with capillary electrophoresis by Agilent 2100 (Bioanalyzer, Agilent Technologies, Santa Clara, CA, United States) and was performed by the UHN Princess Margaret Genomics Centre (MaRS Centre, TMDT, Toronto, ON, Canada).

### RNA Library Preparation and Sequencing (RNAseq)

Both cDNA library generation and RNA sequencing were performed by the Donnelly Sequencing Centre at the University of Toronto (Toronto, ON, Canada) using Illumina’s TruSeq stranded mRNA enrichment for library preparation. cDNA was generated from amplified mRNA, which was purified from total RNA. To purify mRNA, oligoT and 3′ poly A tails were hybridized to mRNA only during transcription in the nucleus. Magnetic beads linked to poly T oligo were used to selectively isolate mRNA. The purified mRNA was then fragmented by chemical shear and size selection was performed to generate mRNA fragments > 100 bps. Fragmented mRNA was reversed transcribed by reverse transcriptase and random primers to generate first strand cDNA. Second strand cDNA generated using DNA polymerase I and RNase H. Adapters were ligated on cDNA fragments, followed by enrichment using PCR to generate the final mRNA-derived cDNA library. For 2 week study samples, HiSeq2500 single-end sequencing was performed at 51 cycles. For 2 day study samples, NextSeq500 single-end sequencing was performed at 75 cycles. Technical replicates were generated by running each sample across 2 lanes to ensure there is no technical variability.

### Analysis of RNA-Sequencing and Microarray Data

For RNA sequencing (RNA-seq), raw sequencing data were processed using the UseGalaxy server (Afgan et al., 2016). Quality control assessments were performed using FastQC version 0.11.7 (Wingett and Andrews, 2018). Technical replicates were concatenated, then aligned to the mus musculus 10 (mm10) reference genome and quantified using Salmon 0.9.1 with default options (Kim et al., 2015; Patro et al., 2017; Srivastava et al., 2019). A gene was considered differentially expressed if the *p*-value was less than 0.05 using Student’s *t*-test. Principal component analysis (PCA) was performed to identify the variance that lies between samples. R script was used for PCA analysis (Ringner, 2008; Jolliffe and Cadima, 2016). An example analysis is shown in Supplementary Figure 1. For our microarray analysis, normalized and processed microarray datasets from sedentary and 6 week exercise left atrial appendages (LAA) were acquired from ArrayExpress (E-MTAB-3106) (Aschar-Sobbi et al., 2015).

Gene Set Enrichment Analysis (GSEA 4.0.3) was used to identify differentially expressed gene sets (*p* < 0.05 and FDR < 0.20) (Subramanian et al., 2005). The C2 curated gene sets (c2 Canonical pathways) and C5 GO gene sets were used for the GSEA analysis. Weighted enrichment statistic was used and Signal2Noise metric was used for ranking genes. Nominal *P*-values of each gene set were given using 10,000 and 1,000 permutations of gene sets for analysis using c2 canonical pathway database and c5 GO database, respectively. Gene sets with fewer than 15 genes or more than 500 genes were excluded. Enrichment maps were generated to visually identify clusters of gene sets on Cytoscape 3.4.0 using the gene sets that were statistically different (*p* < 0.05 and FDR < 0.20) between two groups. Edge and node cut-off values were set to 0.375 (default) and 0.1, respectively. Wordcloud version 3.1.3 was used to annotate clusters. The “difference-of-the-difference” analysis was conducted to identify TNF-dependent pathways by subtracting the gene sets that were significantly differentially regulated between sedentary and swim in WT samples from those differentially regulated between sedentary and swim in KO samples.

Heat maps and hierarchal clustering of genes were performed with MATLAB (version 2016a), using all genes belonging to ECM, focal adhesion, integrin and cell-cell communication- related gene sets. Comparisons of transcripts per kilobase million (TPM) expression for individual genes from RNA-seq between two groups utilized unpaired (two-tailed) *t*-test, and genes with *P*-values of less than 0.05 were considered significant. All genes in the heat maps are significantly different between sedentary and swim WT (*p* < 0.05).

### Telemetric Hemodynamics

Radiofrequency emitting hemodynamic telemetry devices (Data Sciences International, Inc.) were implanted sub-dermally into the interscapularis region. A fluid-filled catheter was inserted into the right common carotid artery and advanced into the left ventricle. After 7 days of recovery, a 30 min baseline recording preceded an acute, 90 min swim exercise bout. Left ventricular end-diastolic pressure (LVEDP) was used as an index of left atrial pressure. Data were analyzed using Ponemah Physiological Platform analysis software (Data Sciences International, Inc.).

### Cardiac Electrical Remodeling and Arrhythmia Vulnerability

Electrical properties and arrhythmia inducibility were assessed as previously described (Aschar-Sobbi et al., 2015). For these measurements, mice were anesthetized (1.5% isoflurane and oxygen mixture) followed by isolation of the right jugular vein and insertion of a 2.0F octapolar recording/stimulation EP catheter (CI’BER Mouse, Numed), which was subsequently advanced into the right ventricle. Programmed electrical stimulations were delivered to the right atria or right ventricle to assess arrhythmia inducibility. All stimulations were delivered at a magnitude of 1.5 times capture threshold and 1 ms pulse duration. Effective refractory periods (ERPs) were determined by delivering nine pulses at 20 ms below the R-R interval followed by an extra stimulation. The S2 coupling interval was initially delivered above capture (∼40 ms) and reduced by 5 ms increments and adjusted until capture was achieved. For arrhythmia induction, 27 pulses at 40 ms intervals were applied to each chamber and reduced at 2 ms decrements to 20 ms. In the absence of inducibility, a second protocol of 20 trains (every 1.5 s) of 20 pulses (2 ms duration) at a 20 ms interpulse interval were applied. Only reproducible episodes of rapid, chaotic, and continuous atrial or ventricular activity of more than 10 s were defined as a sustained arrhythmic event.

### Histology and Macrophage Infiltration

For histology, hearts were perfused with PBS containing 1% KCl followed by 4% paraformaldehyde (PFA) in 0.01 M PBS and stored overnight in 4% PFA in 0.01 M PBS at 4°C. Hearts were then embedded in paraffin and 5 μm thin sections were stained with Picrosirius red (PSR) for collagen visualization and quantification. Collagen expression was quantified using ImageJ software as the ratio of positively stained tissue area to total tissue area of each section using the threshold method (Hadi et al., 2011). To quantify macrophage infiltration, antibodies against mouse Mac-3 (1:200, BD Pharminogen, Cat.#553322) were used with the streptavidin-biotin diaminobenzidine chromogen detection method (Vector Laboratories). Mac-3-positive cells were counted in at least three different left atrial appendage sections (100 μm apart) in each replicate and normalized to the total tissue area of each slice. Images were acquired using Metamorph software (Molecular Devices) and analyzed using ImageJ software.

### *In vitro* ADAM17 Enzymatic Activity Assay

*In vitro* ADAM17 enzymatic activity was measured, as previously described (Shen et al., 2018). Briefly, atrial protein was extracted using a lysis buffer with a high yield of membrane-bound proteins (Cacodylic acid 10mM, NaCl 150 nM, ZnCl2 1 μM, CaCl2 20 mM, NaN3 1.5 mM, Triton X-100 1%, pH 5.0). Mca-Pro-Leu-Ala-Gln-Ala-Val-Dpa-Arg-Ser-Ser-Ser-Arg-NH2 fluorogenic peptide substrate III (R&D Systems, ES003) was used as the substrate for ADAM17. Mca-Pro-Leu-OH (Bachem, M-1975) calibration standard was used to calculate the conversion factor, and recombinant mouse ADAM17 (R&D Systems, 2978-AD) served as a positive control. The ADAM17 activity assay was carried out per the R&D systems protocol. A total amount of 5 μg protein was used for ADAM17 enzymatic activity assay, which was run as a kinetic assay mode for 2 h. Each sample was run in triplicates. ADAM17 activity is expressed as pmol/min/μg tissue protein.

### Gelatin Zymography

MMP2 and MMP9 activity levels were assessed by *in vitro* gelatin zymography, as previously described (Jana et al., 2020). In brief, equal amounts (20 μg) of non-reduced atrial tissue lysate were run on 8% SDS-PAGE gel containing 1 mg/ml gelatin. Following electrophoresis, gels were renatured with 2.5% Triton X-100 buffer for 60 min (room temperature). The gels were then put in calcium assay buffer (50 mM Tris-Cl, pH 7.5, 5 mM CaCl2, 150 mM NaCl) and incubated overnight (37°C). Gels were then stained with 0.05% Coomassie Blue G-250, and grayscale images were scanned and inverted for densitometric quantification. Band intensity was quantified using the inbuilt ImageQuant TL software (Version 7.0 GE Healthcare) and normalized to a loading control.

### Immunofluorescent Analyses

Left atrial tissue from each heart was flash-frozen in mounting compound (OCT). Immunohistochemical staining was performed on 5 μm sections (all other staining). Immunostaining for OPN (ab8448; Abcam), SPARC (MAB941, R&D Systems), and GAPDH (#2118. Cell Signaling Technology) were performed on frozen OCT sections, as previously described (Sakamuri et al., 2016).

### Protein Extraction and Western Blot Analyses

Flash-frozen atria were freeze-crushed in tissue lysis buffer containing EDTA-free protease inhibitor cocktails and processed for protein extraction and immunoblotting as previously described (Sakamuri et al., 2016). Antibodies used for immunoblotting were as follows: OPN (ab8448; Abcam), SPARC (MAB941, R&D Systems), and GAPDH (#2118, Cell Signaling Technology). Band intensities were quantified using densitometry analysis software (ImageQuant TL 7.0; GE) and values were normalized to GAPDH expressions for each sample.

### Statistics

Statistical analyses of transcriptional changes are described above. Unpaired (two-tailed) *t*-test was used to assess differences in ADAM17 activity, gel zymography, and western blot analyses. *P*-values of less than 0.05 were considered significant.

## Results

**FIGURE 1:**
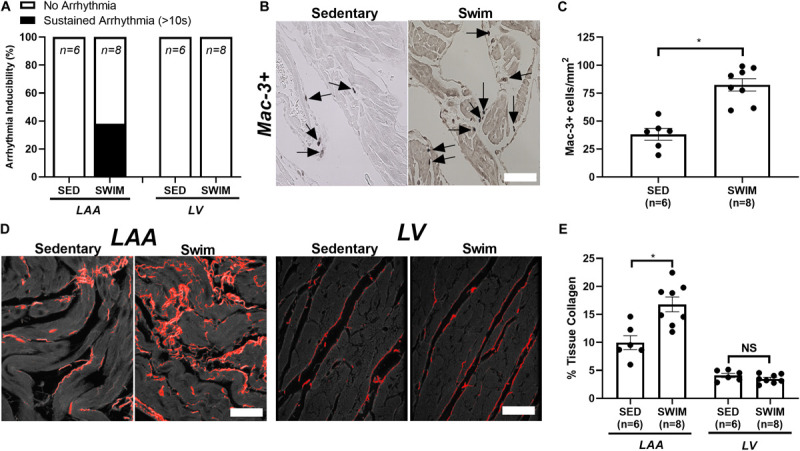
Intense exercise-induced adverse atrial remodeling and atrial fibrillation (AF) vulnerability. **(A)** 6 week swim exercise training is associated with increased atrial fibrillation (AF) inducibility, with no evidence of left ventricular (LV) arrhythmia vulnerability, compared to sedentary mice. **(B,C)** Swim exercise was associated with increased inflammatory cell infiltration (macrophage, Mac-3+) compared to sedentary mice. **(D,E)** Increased fibrosis (%tissue collagen) was observed in the left atrial appendage (LAA) with 6 week swim exercise compared to sedentary mice, with no elevations in fibrosis observed in the LV. Data presented as mean ± SEM. *P < 0.05.

Consistent with previous work (Aschar-Sobbi et al., 2015), 6 weeks of swimming increased (*P* < 0.001) AF inducibility (Figure 1A), which was associated with increased (*P* < 0.003) left atrial (LA) inflammatory (Mac-3+) cell infiltrations (Figures 1B,C) and fibrotic remodeling (Figures 1D,E), as well as hypertrophy (not shown) in left atrial appendages (LAA). By contrast, the left ventricle (LV) showed enhanced function without fibrosis (Figures 1D,E), inflammation (data not shown) nor increased arrhythmia vulnerability in response to exercise, consistent with a chamber-specific effect of intense exercise.

Our previous microarray data from atria of mice after 6 weeks (Aschar-Sobbi et al., 2015) revealed exercise-induced transcriptional changes consistent with increases in inflammatory genes. As shown in Supplementary Figures 2,3, additional bioinformatics analyses verified enrichment in exercised atria of gene sets/clusters associated with inflammation (*P* < 0.05, FDR < 0.2) along with pathways involved in cell cycle regulation, mitochondrial fatty acid, and biological oxidation as well as metabolism/processing of DNA, RNA, and amino acids. Additionally, gene sets linked to mechanosensitive pathways (i.e., focal adhesion kinases (FAK), integrins, cell-cell communication) as well as extracellular matrix (ECM) remodeling (i.e., collagen formation, degradation, biosynthesis, assembly, cross-linking, and matrisome enzymes) were also differentially regulated between WT exercised and sedentary atria. However, despite the elevations in atrial fibrosis after 6 week of exercise, transcriptional levels of individual collagen genes (i.e., *Col1a1*, *Col3a1*, and *Col4a1*) were paradoxically reduced in WT exercised atria.

The unexpected lack of transcriptional elevations in collagen genes, combined with the inability of TNF blockade to prevent atrial fibrosis when started 3 weeks after exercise initiation (Aschar-Sobbi et al., 2015), suggests that pathways driving fibrosis are engaged early following exercise initiation. Therefore, we investigated the transcriptome responses after 2 weeks of exercise. In order to get greater gene coverage, we used deep RNA sequencing (RNA-seq) for these studies. Since TNF gene disruption prevented adverse atrial changes and exercise-induced AF in a chamber-dependent manner, RNA-seq was performed on atria and ventricles from both WT and TNF-KO mice. We first present the transcriptome changes with exercise in atria and discuss the ventricular results thereafter. For clarity, we illustrate our bioinformatic results by displaying each differentially regulated gene set as an individual dot (blue for enriched in swim and red enriched in sedentary). All closely related gene sets were represented by lines using Cytoscape 3.4.0 which allowed gene sets to be grouped into “gene clusters” with common and overlapping function and/or genes. To help focus our discussion, our graphic representations did not include clusters of gene sets with 2 or fewer related gene sets. The total number of differentially regulated gene sets and the number of gene sets in each cluster are presented in each figure.

### Transcriptional Responses in Atria After 2 Weeks of Exercise

Principal component analysis (PCA) showed (an expected) distinct separation between atria and ventricles (Supplementary Figure 1). Surprisingly, there was little separation between WT and TNF-KO, regardless of chamber or exercise status. A remarkable feature of PCA results is the much larger effect of exercise on atrial vs. ventricular transcriptomes, for either genotype. These findings establish that exercise has a far greater impact on atrial vs. ventricular transcriptomes while TNF ablation has a relatively minor impact on exercise-induced changes in either chamber.

**FIGURE 2:**
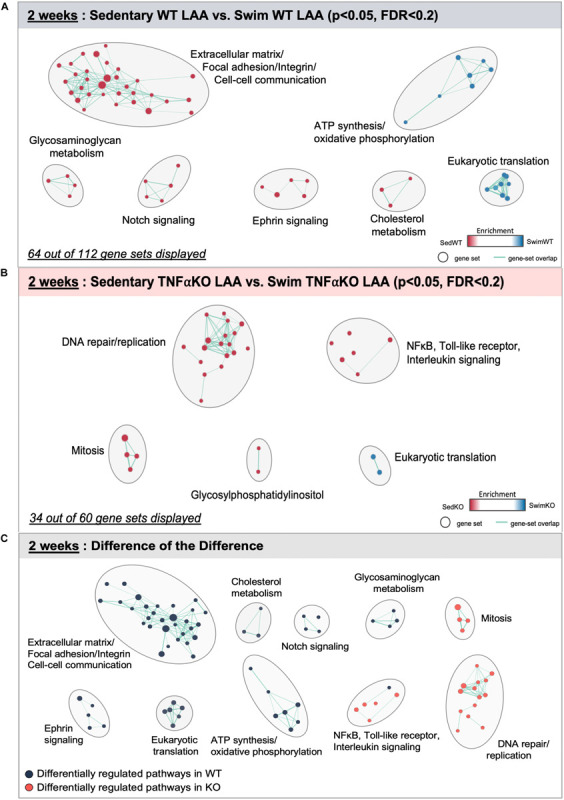
Differentially regulated pathways in the atria of 2 week swim exercised wild-type (WT) and tumor necrosis factor (TNF) knockout (KO) mice. **(A)** Gene set enrichment analysis (GSEA) and enrichment mapping showing clusters of differentially regulated pathways in the left atrial appendage (LAA) between WT 2 week swim (blue dots) and sedentary (red dots) mice. **(B)** Enrichment map showing clusters of differentially regulated pathways in the left atrial appendage (LAA) of TNF-KO 2 week swim and sedentary mice. **(C)** Enrichment map of the difference of the difference analysis revealing clusters of exercise-induced differentially regulated pathways in WT (blue dots) vs. TNF-KO (orange dots) mice. Only gene sets that form clusters are shown for clarity, with connecting lines indicating gene set overlap. Nominal *P*-value < 0.05, false discovery rate (FDR) < 0.20.

The specific gene sets that were differentially affected in WT atria by 2 weeks of exercise are represented in Figure 2 and Supplementary Table 1. The 2 week results in the TNF-KO mice are presented later. These analyses identified 112 differentially regulated (*P* < 0.05 and FDR < 0.2) gene sets, with 64 of these falling into clusters with common function (Figure 2A). Of these, gene sets linked to ATP synthesis and oxidative phosphorylation were enriched with swimming, which is not unexpected given the known cardiac bioenergetic adaptations to exercise (Vega et al., 2017). More interesting perhaps, was the finding that ∼28% of the differentially regulated gene sets were linked to mechanosensing (i.e., integrin/focal adhesion signaling), cell-cell communication, and ECM (i.e., collagen turnover, cross-linking, and matrisome remodeling), compared to only 15% after 6 weeks of exercise. As discussed later, differential regulation of these gene sets seems highly relevant because atrial stretch is central to AF pathogenesis (Vranka et al., 2007; Remes et al., 2008). Nevertheless, these gene sets were enriched in sedentary atria of WT mice (see Supplementary Table 2 for separation of these gene sets into different functional categories). Importantly, the expression levels of the major cardiac collagen types did not vary between the groups, suggesting that fibrotic responses to exercise after 2 weeks are limited to collagen turnover and stability (discussed below). Consistent with this notion, notch as well as the closely related Ephrin-related pathways were also enriched in the atria of WT sedentary compared to swim mice, which both play central roles in early embryogenesis (Sanz-Ezquerro et al., 2017) and are associated with hypertrophy and fibrosis in the heart as well as other tissues (Su et al., 2017).

Despite the protective effects of TNF blockade on the atrial changes induced by exercise, the 2 week WT exercised atria did not show clear evidence of differential regulation of genes related to inflammation or TNF signaling, although several TNF-related pathways were just beyond our cut off criteria [NFκB-IKK (*P* = 0.04, FDR = 0.327), RelA (*P* = 0.06, FDR = 0.329), TNFR1 (*P* = 0.08, FDR = 0.384), and IL1R (*P* = 0.10, FDR = 0.384)]. By contrast, TNF-related gene sets [e.g., Toll-like receptor, interleukin, and TNF-related pathways (NF-κB and p38 MAPK)] were differentially regulated between swim and sedentary atria from TNF-KO mice, with enrichment in the sedentary group (Figure 2B and Supplementary Table 1). It is important to note that the number of gene sets related to ECM/mechanosensing was far less (i.e., 2 vs. 30) and did not form clusters in TNF-KO compared to WT mice, which aligns with the absence of exercised-induced atrial fibrosis when TNF is inhibited. Although at first glance this pattern of differential regulation with exercise in TNF-KO atria seems unexpected, we provide additional data below supporting the conclusion that TNF plays a permissive, rather than a primary role, in exercise-mediated atrial remodeling.

**FIGURE 3:**
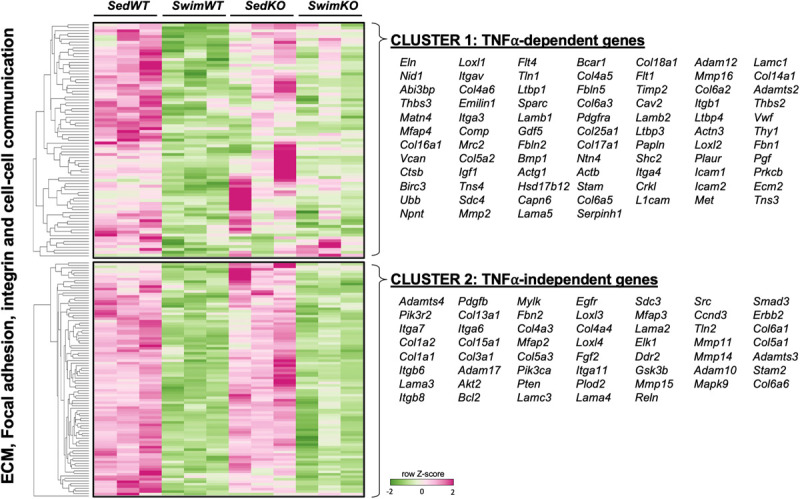
Heat map and clustering analysis of the tumor necrosis factor (TNF)-dependence of extracellular matrix (ECM)-, integrin-, and cell-cell communication-associated genes in 2 week swim exercised mice. Heat map and cluster analysis of individual genes belonging to ECM-, integrin-, and/or cell-cell communication-associated gene sets that are TNF-dependent (cluster 1) or TNF-independent (cluster 2). Note that all individual genes were enriched in sedentary (Sed) vs. swim mice. TNF-dependent (cluster 1) is: SedWT vs. SwimWT (*P* < 0.05) and SedKO vs. Swim KO (not *P* < 0.05). TNF-independent (cluster 2) is: SedWT vs. SwimWT (*P* < 0.05) and SedKO vs. SwimKO (*P* < 0.05).

The TNF-dependence of the gene sets that are differentially regulated with exercise are summarized in Figure 2C as the “difference-of-the-difference” results (see section “Materials and Methods”). These analyses reveal, not unexpectedly, that exercise induces TNF-dependent changes in gene sets involved with ECM/mechanosensing, collagen production/turnover, fatty acid metabolism, oxidative phosphorylation as well as notch and ephrin signaling, with all these gene sets being enriched in the atria of WT compared to TNF-KO mice. To better understand the involvement of TNF in exercise-induced atrial remodeling, we further assessed the TNF-dependence of specific differentially regulated genes by generating heat maps of genes related to mechanotransduction and ECM (Figure 3). For these purposes, genes were separated into TNF-dependent (cluster 1 genes, whose expression differed in WT only) vs. TNF-independent genes (cluster 2 genes whose expression differ in both genotypes). As shown in Figure 3, far more genes were regulated in a TNF-dependent than a TNF-independent manner. Since TNF-KO abrogates atrial fibrosis as well as AF inducibility, we focused our attention initially on TNF-dependent collagen/ECM/mechanosensing genes (Supplementary Table 2). Of these, *Mmp2* and *Mrc2* are reduced in atria after 2 weeks of exercise which is of particular interest because these genes are also reduced in the age-related fibrosis of multiple tissues (Podolsky et al., 2020) and AF is strongly linked to aging (Heeringa et al., 2006).

It is also worth stating that many specific ECM/mechanosensing-related genes whose expression was downregulated in TNF-dependent manner with exercise have been linked previously to AF and ECM remodeling, including *Comp* (Zou et al., 2018; Thomas et al., 2019), *Thbs2* (Yang et al., 2000), *Ltbp1* and *Fbn1* (Zhang et al., 2020; Figure 3 and Table 1). On the other hand, most genes linked to collagen production (i.e., *Col1a1*, *Col1a2*, *Col3a1*) were TNF-independent. Since TNF-KO abrogates fibrosis and AF inducibility, these results suggest (see section “Discussion”) that the cluster 2 genes are not central to the adverse atrial changes induced by exercise. In light of the impact of TNF on atria induced by exercise, it is worth pointing out that only two genes, *Comp* (TNF-dependent) and *PIK3R2* (TNF-independent) are upregulated with exercise in swim WT vs. swim TNF-KO atria (see Discussion). Additional gene sets and individual genes linked to ECM/mechanosensing and/or AF are listed in Table 1 and Supplementary Tables 1–3, respectively.

**TABLE 1:** Differentially regulated genes with 2 weeks exercise of the ECM-receptor, integrin, and cell-cell communication pathways associated with hypertrophic remodeling and/or atrial fibrillation (AF).

**Gene**	**Gene product**	**Directional change with swim exercise**	**Tumor necrosis factor (TNF) dependent?**	**Link(s) to fibrotic and/or hypertrophic remodeling and AF vulnerability**
*Adam10*	A disintegrin and metalloprotease (ADAM) 10	↓	No	Processes membrane bound TNF to a soluble form, which in turn can induce MMPs and drive ECM remodeling (Manso et al., 2006; Murphy, 2008)
*Adam12*	A disintegrin and metalloprotease (ADAM) 12	↓	Yes	Regulator of MMPs and linked to prevention of cardiac hypertrophic and fibrotic remodeling through reductions in TGF-β- and integrinβ1-mediated FAK/Akt, ERK, and Smad signaling (Frangogiannis, 2012; Nakamura et al., 2020)
*Adam17*	A disintegrin and metalloprotease (ADAM) 17	↓	No	A metalloprotease and disintegrin, also known as TNF-α converting enzyme (TACE), that cleaves TNF to its soluble form and has been implicated in pressure overload-induced hypertrophic and fibrotic remodeling (Xu et al., 2016) and AF vulnerability (Weeke et al., 2014)
*Adamts2*	Adamts2	↓	Yes	Extracellular enzyme that activates pro-collagens I, II, III, and V (Wang et al., 2017b), and is a regulator of MMPs linked to cardiac hypertrophic and fibrotic remodeling in pressure-overload (Dong et al., 2013)
*Bcl2*	B-cell lymphoma 2	↓	No	Increased expression of BCL-2/BCL-2-associated X protein (BAX) linked to fibrosis and apoptosis in AF (Xu et al., 2013; Diao et al., 2016)
*Bmp1*	Bone morphogenetic protein 1	↓	Yes	A peptidase that cleaves the C-terminal pro-peptides of procollagen I, II, and III and mediates the proteolytic activation of lysyl oxidase LOX (Rodriguez and Martinez-Gonzalez, 2019)
*Col4a4, Col5a1, and Col5a3*	Collagen type IV, alpha 4 chain; collagen type V, alpha 1 chain; and collagen type V, alpha 3 chain	↓	No	Collagen transcripts linked to AF and rhythm outcome following ablation (Husser et al., 2016)
*Col6a6*	Collagen type VI Alpha 6 Chain	↓	No	Collagen protein encoding gene containing multiple von Willebrand factor (vWF) domains. Linked to extracellular matrix (ECM)-receptor interactions and is upregulated in AF (Zou et al., 2018)
*Ctsb*	Cathepsin B	↓	Yes	Lysosomal cysteine proteases localized in lysosomes and endosomes that function to degrade cellular substrates (Turk et al., 2000). Stimulated by TNF and linked to hypertrophic and fibrotic cardiac remodeling (Cheng et al., 2012b). Upregulated in AF (Thomas et al., 2019)
*Erbb2*	HER-2; receptor tyrosine kinase	↓	No	A receptor involved in physiological cardiac adaptations and hypertrophic remodeling through the activation of MAPK, PI3K/Akt and Src/FAK signaling pathways (Vermeulen et al., 2016)
*Fbln2*	Fibulin-2	↓	Yes	ECM glycoprotein involved in angiotensin-II-mediated TGF-β signaling and cardiac hypertrophy (Zhang et al., 2014)
*Fbn1*	Fibrillin 1	↓	Yes	Large ECM glycoprotein and constituent of the myocardial ECM that is linked to reactive and reparative fibrotic remodeling (Bouzeghrane et al., 2005) and is upregulated in AF (Zhang et al., 2020)
*Igf1*	Insulin-like growth factor-1	↓	Yes	A hormone that has pleiotropic actions in the heart and mediated eccentric hypertrophy through PI3K- and MAPK-dependent mechanisms (Lavandero et al., 1998)
*Itga4, Itgb1, Itgb6*	Integrin alpha-4, beta-1, and beta-6 precursors	↓	No	ECM proteins involved in mechanotransduction linked to AF incidence and rhythm outcome following ablation (Husser et al., 2016)
*Ltbp1*, *Ltbp3*, and *Ltbp4*	Latent transforming growth factor (TGF)- β binding proteins	↓	Yes	Family of secreted multidomain proteins that bind to and regulate TGFβ-dependent activation and pro-fibrotic remodeling (Goumans and Ten Dijke, 2018) as well as AF (Thomas et al., 2019; Zhang et al., 2020)
*Mfap4*	Microfibril-associated protein 4	↓	Yes	A matricellular protein associated with AF and atrial fibrotic remodeling (Zhang et al., 2019)
*Pgf*	Placental growth factor	↓	Yes	A member of the VEGF (vascular endothelial growth factor) family known to induce cardiac fibroblasts to secrete TNF and other pro-hypertrophic factors (Accornero and Molkentin, 2011)
*Pdgfb*	Platelet-derived growth factor subunit B	↓	No	Increased PDGF-B expression linked to focal cardiac fibrosis and moderate cardiac hypertrophy (Gallini et al., 2016)
*Pdgfra*	Platelet-derived growth factor α receptor	↓	Yes	Cardiac mast cells synthesize and release PDGF-A and mediates both fibrosis and AF in pressure-overloaded hearts (Liao et al., 2010; Wang et al., 2017a)
*Plod2*	Procollagen-lysine, 2-oxoglutarate 5-dioxygenase 2 (PLOD2)	↓	No	Lysyl hydroxylase linked to both TNF- and TGFβ1-mediated pyridinoline cross-linking inherent to fibrotic cardiac remodeling (van der Slot et al., 2005)
*Pten*	Phosphatase and tensin homolog (PTEN)	↓	No	A protein linked to increased pathological hypertrophy and progression to heart failure in response to biomechanical stress (Oudit et al., 2008)
*Reln*	Reelin	↓	No	A large secreted ECM glycoprotein that regulates pathways associated with ECM-receptor interaction and focal adhesion and its expression is linked to AF (Husser et al., 2016; Zhao et al., 2018; Zou et al., 2018)
*Sdc4*	Syndecan-4	↓	Yes	A transmembrane (type I) heparan sulfate proteoglycan that interacts with the ECM and is a primary determinant in collagen cross-linking and LOX induction in pressure-overload hearts (Herum et al., 2015)
*Serpinh1*	Heat shock protein 47 (HSP47)	↓	Yes	A chaperone protein for collagen involved in cardiac injury-induced fibrosis and reductions in hypertrophy in cardiac pressure-overload (Khalil et al., 2019)
*Sparc*	Secreted protein acidic and cysteine rich (Sparc)	↓	Yes	A matricellular protein that is activated by several MMPs (i.e., MMP-2) and is elevated in cardiac hypertrophy and fibrosis in pressure-overload (Bradshaw et al., 2009) and aging (Bradshaw et al., 2010)
*Src*	Proto-ongogene tyrosine-protein kinase Src	↓	No	A tyrosine kinase that phosphorylates FAK (Frame et al., 2010) and is activated in response to integrin clustering and activation as well as LOX-mediated ECM remodeling (Schneider et al., 2008)
*Thbs2*	Thrombospondin-2	↓	Yes	A matricellular protein involved in myocardial matrix integrity (Schroen et al., 2004) and linked to protection against inflammatory-induced cardiac injury and dysfunction (Papageorgiou et al., 2012)
*Tln1*	Talin-1	↓	Yes	A ubiquitously expressed protein localized to costameres that become more prominent during mechanical stress-induced cardiac hypertrophy and fibrosis (Manso et al., 2013) and is upregulated in AF (Weeke et al., 2014)
*Vwf*	von Willebrand factor	↓	Yes	A prothrombotic plasma marker and index of endothelial damage and dysfunction that is prominently linked to AF incidence, rhythm outcome following ablation (Husser et al., 2016) and stroke risk (Zhong et al., 2018; Ye et al., 2020)

### Transcriptional Responses in Atria After 2 Days of Exercise

The observation that ECM/FAK/integrin gene sets pathways were generally enriched in sedentary atria at 2 and 6 weeks, despite atrial fibrosis at 6 weeks in exercised mice, prompted us to perform RNAseq measurements in hearts after only 2 days of exercise (i.e., 4-sessions of 90 min swims). Consistent with 2 week data, PCA showed the expected separation between atria and ventricles (Supplementary Figure 1). Surprisingly, while there were distinct separations between exercise and sedentary atrial samples, this was not true in ventricles, suggesting a much smaller effect of exercise on ventricular transcriptomic remodeling. Moreover, there was overlap between WT and TNF-KO, regardless of chamber or exercise status, suggesting TNF ablation may have a minor impact on acute exercise-induced changes.

**FIGURE 4:**
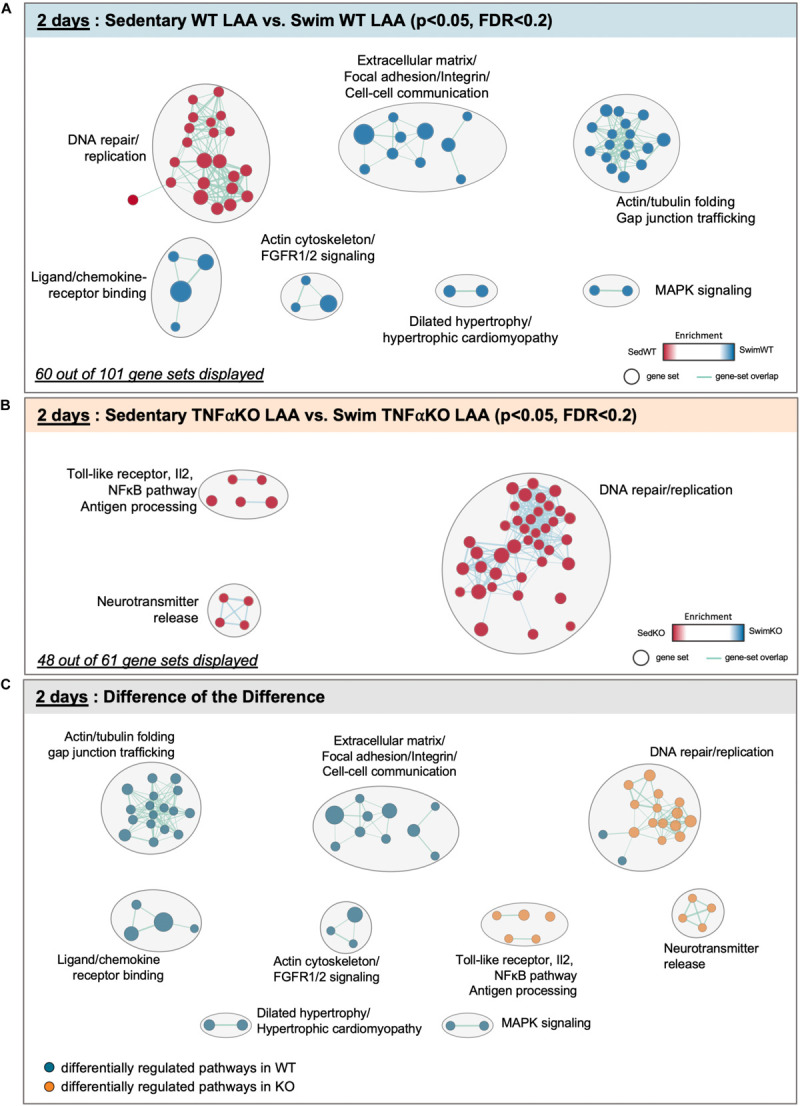
Differentially regulated pathways in the atria of 2 day swim exercised wild-type (WT) and tumor necrosis factor (TNF) knockout (KO) mice. **(A)** Gene set enrichment analysis (GSEA) and enrichment mapping showing clusters of differentially regulated pathways in the left atrial appendage (LAA) between WT 2 day swim (blue dots) and sedentary (red dots) mice. **(B)** Enrichment map showing clusters of differentially regulated pathways in the LAA of TNF-KO 2 day swim (blue dots) and sedentary (red dots) mice. **(C)** Enrichment map of the difference of the difference analysis revealing clusters of exercise-induced differentially regulated pathways in WT (blue dots) vs. TNF-KO (orange dots) mice. Only gene sets that form clusters are shown for clarity, with connecting lines indicating gene set overlap. Nominal *P*-value < 0.05, false discovery rate (FDR) < 0.20.

The results of our GSEA analyses for mice after 2 days of exercise are summarized in Figure 4 and Supplementary Table 4. The data reveals that 101 gene sets were differentially regulated (*P* < 0.05, FDR < 0.2) between swim (total of 61 differentially regulated gene sets) vs. sedentary (40 gene sets) atria in WT mice, with 60 sets clustering into common pathways (Figure 4A). Importantly, unlike what was seen after 2 and 6 weeks of exercise, ECM/mechanosensing gene sets were now more enriched in exercise vs. sedentary atria (Supplementary Table 4). Acute exercise also induced enrichment in gene sets related to actin/tubulin folding, which cross-talks to many hypertrophic signaling pathways (i.e., MAPK/FGFR1/2) linked to dilated and hypertrophic cardiomyopathy (Caporizzo et al., 2019), as well as IQGAPs, PAKs, and AMPK activation (see section “Discussion”) (Hedman et al., 2015; Daskalopoulos et al., 2016). On the other hand, TNF-related gene sets in acute exercise were only differentially regulated in TNF-KO atria (Figure 4B) with reductions in many specific pro-inflammatory genes in exercised mice [i.e., toll-like receptor 10 (*p* < 0.008, FDR = 0.178), IL-2 (*p* = 0.018, FDR = 0.187), IL-2-STAT5 (*p* = 0.018, FDR = 0.240), TAK1 (*P* < 0.037, FDR = 0.278), and NFκB (*P* < 0.005, FDR = 0.124)].

**FIGURE 5:**
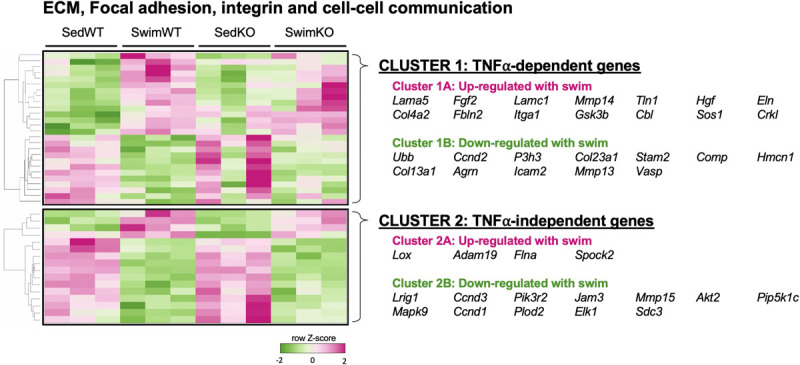
Heat map and clustering analysis of the tumor necrosis factor (TNF)-dependence of extracellular matrix (ECM)-, integrin-, and cell-cell communication-associated genes in 2 day swim exercised mice. Heat map and cluster analysis of up- or down-regulation of individual genes belonging to ECM-, integrin-, and/or cell-cell communication-associated gene sets that are TNF-dependent (cluster 1) or TNF-independent (cluster 2) in 2 day swim mice. TNF-dependent (cluster 1) is: SedWT vs. SwimWT (*P* < 0.05) and SedKO vs. Swim KO (not *P* < 0.05). TNF-independent (cluster 2) is: SedWT vs. SwimWT (*P* < 0.05) and SedKO vs. SwimKO (*P* < 0.05).

The difference-of-the-difference analyses after 2 day exercise clearly establish that gene sets involved in mechanosensitive pathways are uniquely differentially enriched in exercised WT atria, while TNF-related signaling and DNA replication/repair pathways are uniquely enriched in sedentary KO mice (Figure 4C). Heat maps of differentially (*P* < 0.05) regulated genes revealed (Figure 5) a distinct pattern after 2 days of exercise compared to 2 weeks. Now, many TNF-dependent genes linked to ECM remodeling as well as AF are upregulated with exercise in WT atria compared to sedentary, including *Mmp14* (Simmers et al., 2016), *Fgf2* (i.e., fibroblast growth factor 2, which activates p38 and induces cardiac hypertrophy as well as fibrosis) (Itoh and Ohta, 2013), *Gsk3*β (Sugden et al., 2008), and *Tln1* (Manso et al., 2013). On the other hand, only *Fgf2* was upregulated (*P* = 0.0142) in WT vs. KO exercised atria, suggesting TNF-dependent activation of FGF signaling pathways may be important early responses that regulate exercise-induced atrial remodeling. Nonetheless, these results suggest that acute exercise leads to TNF-dependent and TNF-independent transcriptome changes affecting ECM remodeling. Additional genes and their links to ECM/mechanosensing and/or AF are listed in Table 2.

**TABLE 2:** Differentially regulated genes with acute (2 day) exercise of the ECM-receptor, integrin, and cell-cell communication pathways associated with hypertrophic remodeling and/or atrial fibrillation (AF).

**Gene**	**Gene product**	**Directional change with swim exercise**	**Tumor necrosis factor (TNF) dependent?**	**Link(s) to fibrotic and/or hypertrophic remodeling and atrial fibrillation**
*Ccnd3*	Cyclin D3	↓	No	A protein shown to be upregulated during hypertrophic growth (Busk et al., 2002) and linked to the pathogenesis of AF (Li et al., 2019)
*Elk1*	Elk-1	↓	No	A transcription factor that is phosphorylated via MEK/ERK kinases and plays a role in cardiac hypertrophy (Babu et al., 2000) as well as regulating stretch-mediated atrial natriuretic factor (ANF) expression (Mahida, 2013)
*Fgf2*	Fibroblast growth factor 2	↑	Yes	A multifunctional polypeptide that is upregulated by stress (Kardami et al., 2004) and involved in p38 MAPK-mediated cardiac hypertrophy (Tanaka et al., 1999) as well as fibrosis (Itoh and Ohta, 2013)
*Gsk3*β	Glycogen synthase kinase 3 beta	↑	Yes	An enzyme that serves as a hub for the regulation of both physiological and pathological hypertrophic and fibrotic remodeling (Lal et al., 2015) via TNF-related pathways (Sugden et al., 2008)
*Itga1*	Integrin, alpha 1	↑	Yes	Cell-cell and cell-matrix adhesion (ECM-receptor interactions), upregulated in AF and linked to rhythm outcome of AF catheter ablation (Husser et al., 2016)
*Lama5*	Laminin subunit alpha-5	↑	Yes	A component of the ECM, specifically basement membranes, that is upregulated in AF (Husser et al., 2016)
*Mmp13*	Matrix metalloproteinase 13	↓	Yes	A matrix metalloproteinase and collagenase that targets collagens I and III and is linked to cardiac hypertrophy and fibrosis in pressure overload hearts (Spinale, 2002)
*Plod2*	Procollagen-lysine, 2-oxoglutarate 5-dioxygenase 2 (PLOD2)	↓	No	Lysyl hydroxylase linked to both TNF- and TGFβ1-mediated pyridinoline cross-linking inherent to fibrotic cardiac remodeling (van der Slot et al., 2005)
*Tln1*	Talin-1	↑	Yes	A protein mediating cell-cell adhesion linked to AF (Weeke et al., 2014) and becomes more prominent at costameres during mechanical stress and modulates hypertrophic and fibrotic remodeling (Manso et al., 2013)

### Transcriptional Changes in Ventricles With Exercise

As mentioned, after 2 weeks of exercise, PCA showed relatively small effects of exercise on ventricles (compared to atria) at all-time points. Before directly comparing LV and LA transcriptomic remodeling, we present the effects of exercise on LV genetic plasticity. After 2 weeks, genes sets associated with oxidative phosphorylation and ribosome translation were enriched in exercised WT mice while the genes sets related to ECM/mechanosensing (as in atria) and cardiomyopathy (i.e., HCM, DCM, ARVC), as well as notch, ephrin/Rho GTPases, and MAPK signaling were enriched in sedentary atria (Supplementary Figure 4A). By comparison, TNF-KO mice showed enrichment of gene sets linked to amino acid metabolism and TCA cycle with exercise while gene sets associated with ECM/mechanosensing, chemokine, interleukin, and T-cell/B-cell receptor pathways were enriched in the sedentary group (Supplementary Figure 4B). Interestingly, after performing the difference-of-the-difference analyses (Supplementary Figure 4C) the majority of gene sets that remained were related to TNF-mediated signaling with differential regulation in TNF-KO mice, suggesting that TNF also serves a role in LV remodeling with exercise, albeit less than in LA.

**FIGURE 6:**
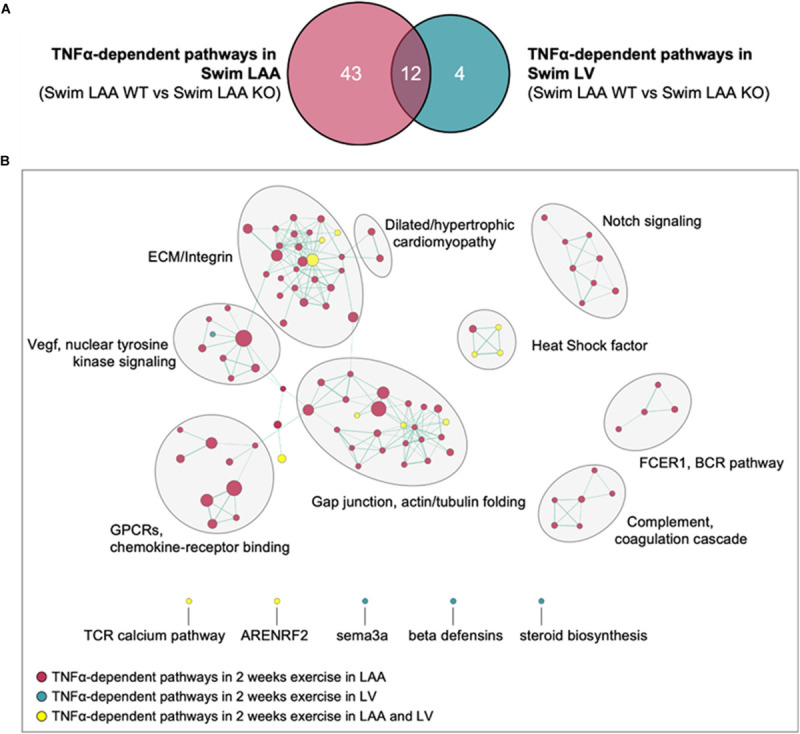
Differential roles of TNF in exercise-induced transcriptomic remodeling with 2 week swim exercise in the left atrial appendage (LAA) vs. left ventricle (LV). **(A)** Venn diagram of exercise- and TNF-dependent (i.e., Swim WT vs. swim KO) differentially regulated gene sets in the LAA vs. LV. **(B)** Gene set enrichment analysis (GSEA) and enrichment mapping showing clusters of TNF-dependent differentially regulated gene sets in the LAA (red dots) and LV (green dots). Gene sets that were TNF-dependent and differentially regulated in both LAA and LV are indicated by yellow dots. Only gene sets that form clusters are shown for clarity. Nominal *P*-value < 0.05, false discovery rate (FDR) < 0.20.

The results above establish that the gene sets inked to ECM/mechanosensitive pathways are quite similar after 2 weeks of exercise between the LA and LV (i.e., Figure 2C and Supplementary Figure 4C), even though the exercise-induced remodeling is different between the chambers. To more directly assess the differential effects of exercise and TNF on chamber-specific transcriptomic remodeling at 2 weeks, a modified difference-of-the-difference analysis was performed in which TNF-dependent pathways were determined by subtracting the gene sets that were significantly differentially regulated between swim in WT and KO samples within each chamber from those differentially regulated between LA and LV samples. As shown in Figure 6A, the number of TNF-dependent differentially regulated gene sets with exercise is far smaller for LVs (i.e., 4) vs. LAs (i.e., 43) at 2 weeks, with little overlap between the gene sets between chambers (i.e., 12). Indeed, while ECM/mechanosensitive pathways were enriched in both the LAs and LVs of sedentary mice at 2 weeks, direct comparisons between chambers highlight the predominance of TNF-dependent differentially gene sets (Figure 6B), including ECM/integrin signaling, dilated/hypertrophic cardiomyopathy, and actin/tubulin folding, in the atria, which reinforces the chamber-specific effects of both exercise and TNF on transcriptome remodeling.

The differential impact of exercise on the LA and LV is also apparent in 2 day acutely exercised mice. Indeed, while we found clear evidence of ECM/mechanosensitive pathway enrichment in WT mice at 2 days in the atria (discussed above), when we assessed ventricular changes after 2 day acute exercise, only 16 gene sets were differentially regulated in WT LVs with most of the pathways linked to cell cycle and DNA replication processes (Supplementary Figure 5A). By contrast, exercised LV from TNF-KO mice had far greater numbers (211) of differentially regulated gene sets, including ECM/integrin, gap junction and actin/tubulin folding, as well as cell cycle and DNA repair/replication (Supplementary Figure 5B), all of which were enriched in sedentary mice. The difference-of-the-difference analysis (Supplementary Figure 5C) confirmed enrichment of the above pathways in TNF-KO compared to WT mice. Indeed, heat map and cluster analysis (Supplementary Figure 6) identified only three genes linked to ECM/mechanosensing, *Mmp16*, *Dst*, and *Reln*, that were upregulated in a TNF-dependent manner with acute swim, compared to the 14 genes identified in the LA. The absence of enrichment of gene sets linked to ECM/mechanosensitive pathways in the LV and their upregulation in the LA with 2 day acute exercise supports our contention that strain-dependent signaling mediates exercise-induced atrial remodeling.

### Acute Effects of Exercise on Atrial Pressures and Collagen Metabolism/Remodeling

**FIGURE 7:**
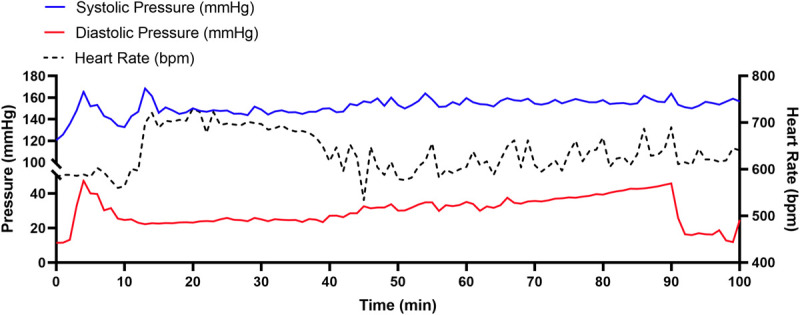
Representative left ventricular (LV) hemodynamic changes measured by implantable pressure-telemetry during an acute swim bout in mice. During a 90 min swim bout, LV diastolic filling pressures (red) increase rapidly from a baseline of ∼10 mmHg to ∼45 mmHg within the first 10 min. Thereafter, the pressure falls to ∼20 mmHg, only to rise steadily to ∼40–45 mmHg by the end of the 90 min swim.

Since AF is primarily observed in cardiovascular conditions associated with elevated diastolic filling pressures (De Jong et al., 2011), we previously postulated (Aschar-Sobbi et al., 2015) that elevated filling pressures seen with exercise may explain the increased incidence of AF in endurance athletes. Consistent with this conjecture, we found that diastolic filling pressures increase from 10 mmHg to ∼45 mmHg within the first 10 min after mice begin swimming exercise (Figure 7). Thereafter the pressure falls to ∼20 mmHg over the next 30–50 min after which the filling pressure steadily rise to 40–45 mmHg after 90 min. Such increases in venous filling pressures would be expected to preferentially stretch the thin-walled and compliant atria, which may explain our observation of mechanosensitive and compensatory hypertrophic pathways being disproportionately activated in LAs compared to LVs.

Taken together, our findings demonstrate prominent time-dependent transcriptional changes in genes related to strain-dependent pathways in response to exercise. However, despite the induction of fibrosis by exercise, the absence of increased collagen expression at all-time points following exercise led us to explore the potential contribution of factors that have previously been shown to mediate post-translational changes in collagen maturation and deposition in the heart. For these studies, we used the 2 day mice and made the measurements 2 h following the final 90 min exercise bout, consistent with our RNA-seq measurements. Given that soluble TNF is required for exercise-induced atrial changes (Lakin et al., 2019), we first measured the activity of TNF-converting enzyme (TACE, or ADAM17), which is upregulated with mechanical stretch and releases active (soluble) TNF (Zhan et al., 2007). Indeed, TACE activity tended to be increased (*P* = 0.211) with swim (142 ± 4 pmol/min/μg, *n* = 5) compared to sedentary mice (129 ± 8 pmol/min/μg, *n* = 6), suggesting activation. It is conceivable that earlier assessment would have displayed even greater TACE activity since we previously found upregulation of TNF-dependent p38 MAPK signaling within 10 min post-exercise (Aschar-Sobbi et al., 2015). We also measured MMP2 and MMP9 activity since these are increased with atrial stretch as well as in CV disease (Yabluchanskiy et al., 2013). We found that pro-MMP2 activity was increased (*P* = 0.0003) while pro-MMP9 activity tended (*P* = 0.098) to be increased in atria after swim completion in 2 day swim compared to sedentary mice (Supplementary Figure 7A), establishing increased collagen turnover with acute exercise.

Since previous studies reported increases in matricellular proteins that mediate post-synthetic collagen turnover in several models (Frangogiannis, 2012; McDonald et al., 2018), we also measured osteopontin (OPN) and SPARC expression levels. Although SPARC expression in atria was unaffected by acute exercise (*P* = 0.562), OPN was decreased (*P* = 0.006), suggesting these matricellular proteins contribute minimally to adverse atrial remodeling in the early response to exercise.

**FIGURE 8:**
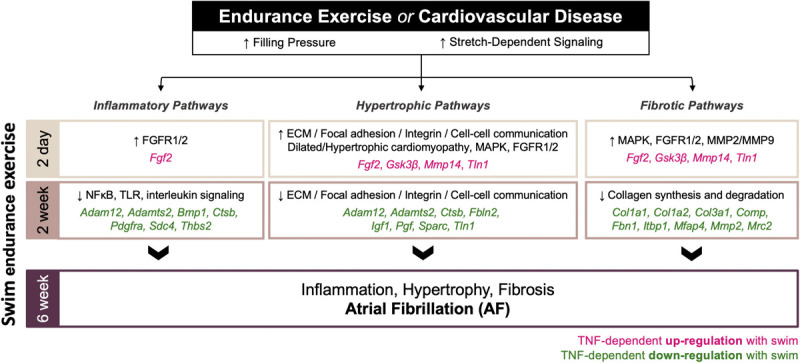
Schematic overview of the time-dependent and atrial-specific exercise-induced tumor necrosis factor (TNF)-dependent transcriptional responses to elevated venous filling pressures and atrial stretch mediating adverse atrial remodeling (inflammation, fibrosis, hypertrophy) and atrial fibrillation (AF) vulnerability.

A schematic overview of the time-dependent and atrial-specific exercise-induced TNF-dependent transcriptomic changes mediating adverse atrial remodeling and AF vulnerability in response to increased filling pressures and atrial stretch are shown in Figure 8.

## Discussion

The transcriptional changes in WT atria were generally similar after 2 vs. 6 weeks of exercise, with about 50% of the differentially regulated gene sets related to ECM and mechanosensing enriched in sedentary mice. We believe that changes in gene sets associated with ECM/mechanosensitive pathways at these time points are relevant because elevated venous filling pressures (i.e., preloads) are seen invariably in virtually all AF conditions (Vranka et al., 2007; Remes et al., 2008), including intense exercise (Reeves et al., 1990; Aschar-Sobbi et al., 2015). Such elevations in filling pressure are expected to favor atrial vs. ventricular stretch and thereby preferentially driving stretch-dependent atrial remodeling (De Jong et al., 2011). This would help explain the appearance of fibrosis in atria but not ventricles. Moreover, the number of differentially regulated genes/gene sets related to ECM/mechanosensing were ∼twofold greater after 2 weeks vs. 6 weeks of exercise suggesting that the atrial responses to exercise adapt and diminish with time, as expected with the appearance of fibrosis. A puzzling finding, however, is the absence of increased collagen mRNA levels in exercised atria at either time point, despite the fibrosis after 6 weeks of exercise. This was surprising because fibrosis seen in most cardiac conditions (i.e., heart failure, hypertension, and cardiomyopathies) is associated with elevated collagen mRNA (Frangogiannis, 2019). The absence of elevated collagen mRNA in fibrotic exercised atria is similar to the discordant pattern seen with age- (Horn and Trafford, 2016; Podolsky et al., 2020) and stress-related (Kandalam et al., 2011) fibrosis in several tissues. As discussed below, we believe these findings have important implications on the mechanisms underlying adverse atrial changes and AF, regardless of the inciting factors.

Consistent with the TNF-dependence of adverse atrial changes with exercise, TNF- and inflammation-related gene sets were differentially regulated after 2 weeks of exercise (similar to 6 weeks of exercise) with the number of differentially regulated gene sets being ∼twofold less in TNF-KO vs. WT atria. On the other hand, after 2 weeks of exercise atria from sedentary TNF-KO mice, but not WT mice, showed enrichment in TNF-related inflammatory gene sets. Since adverse atrial changes do not occur in TNF-KO mice, it appears that TNF plays a permissive role in exercise-induced inflammation rather than being a primary factor. By contrast, the primary exercise-induced gene changes in TNF-KO atria are related to DNA replication and repair, whose significance will require further studies. Collectively, the differences in atrial transcriptome remodeling after 2 weeks of exercise are consistent with the pleiotropic actions of TNF (Tracey and Cerami, 1994).

To gain insight into possible mechanisms underlying the TNF-dependent atrial changes induced by 2 weeks of exercise, we examined transcript levels of individual genes between the 4 groups. Consistent with our enrichment maps, the bulk of the differentially regulated genes related to ECM/mechanotransduction had lower expression levels in exercised atria of both WT and KO mice. Moreover, most of these genes have been linked to increased tissue fibrosis, despite the absence of atrial fibrosis and AF inducibility in exercised TNF-KO mice, which suggests that many of these gene changes are likely of limited relevance in driving exercise-induced fibrosis (Chou et al., 1996; Sivasubramanian et al., 2001). However, it is noted in previous studies that reductions in the metalloproteinase, *Mmp2*, and the canonical collagen endocytic receptor (Engelholm et al., 2003), *Mrc2* (which are both TNF-dependent genes that are reduced in WT exercised atria), are linked to age-dependent fibrosis (Podolsky et al., 2020). These TNF-dependent changes are particularly interesting since atrial fibrosis and AF are generally seen with aging (Gramley et al., 2009; Ravassa et al., 2019), and AF is especially prevalent in veteran endurance athletes (Karjalainen et al., 1998; Mont et al., 2002). Also, these *Mmp2* reductions paralleled reductions in *Mmp14*, albeit independently of TNF, which has been associated with AF (Simmers et al., 2016) and shown to activate MMP-2 (Jr and Nagase, 2000). On the other hand, when we directly compared WT and TNF-KO swim atria, the majority of genes were increased in exercised atria from TNF-KO relative to WT, with the only gene that was increased in a TNF-dependent manner after 2 weeks of exercise in WT atria was *Comp*, which is a biomarker for cardiac fibrosis and hypertrophic remodeling (Huang et al., 2013; Zhao et al., 2018). *Comp* is involved in non-collagen ECM-receptor interaction (Rosenberg et al., 1998) and appears from multiple studies to contribute to the pathogenesis of AF (Zou et al., 2018; Thomas et al., 2019).

As the ECM/mechanosensitive pathways did not show enrichment at 2 and 6 weeks in our exercised mice, we also performed RNAseq measurements after only 2 days of exercise. Importantly, at this time point gene sets associated with ECM/mechanosensitive as well as hypertrophic signaling pathways were enriched in exercised atria from WT mice, which would appear to align with the elevated filling pressures seen in exercise and AF-related conditions (Reeves et al., 1990; De Jong et al., 2011; Aschar-Sobbi et al., 2015). Of particular note is the enrichment of tubulin folding and MAPK pathways in exercised WT (but not TNK-KO) atria. Tubulin assembly/disassembly in microtubules is involved in mechanosensing (White, 2011) in a number of cell types and is interdependent on MAPKs (Samaj et al., 2004), particularly p38 kinases (Ramkumar et al., 2018). These results suggest that strain-dependent signaling via microtubule assembly/disassembly may play a role in driving early atrial responses to atrial stretch occurring during exercise, possibly in concert with the recruitment of TNF-dependent transduction, consistent with TNF’s mechanosensing properties (Kroetsch et al., 2017). Microtubule involvement is consistent with the pioneering studies by George Cooper (4th) who showed that p21-activated kinase-1 (Pak1)-dependent microtubule assembly plays a central role in the early response to pressure overload and mechanical stretch in right ventricular cardiomyocytes (Cheng et al., 2012a). Indeed, Pak1 regulates exercise-induced cardiac hypertrophy (Davis et al., 2015), which aligns nicely with our 2 day atrial analyses showing exercise-induced upregulation of *Flna* (filamen A), a cytoprotective protein that is upregulated with mechanical stress (D’Addario et al., 2003) and is essential for actin/cytoskeletal dynamics (Vadlamudi et al., 2002) through interdependent p38- (D’Addario et al., 2002) and Pak1-mediated signaling (Zhang et al., 1995; Shifrin et al., 2012). We also found enrichment of other pathways, including IQGAPs and AMPKs, in acutely exercised WT atria which are involved in the early compensatory responses to pressure overload stimuli that may be harbingers of fibrotic remodeling in the long-term (Hermida et al., 2013; Hedman et al., 2015; Daskalopoulos et al., 2016). Taken together, these observations suggest that the loss of TNF leads to an inhibitory modulation of mechanosensitive signaling pathways which is consistent with the stretch-dependence of TNF activation (Kroetsch et al., 2017) and the regulation of FAK by TNF (Funakoshi-Tago et al., 2003; Murphy et al., 2019) via MAPK signaling and IL-6 expression (Schlaepfer et al., 2007). Given the absence of exercise-induced adverse atrial remodeling and AF with TNF inhibition, our results suggest stretch-activation of TNF may tip the scales toward maladaptive compensatory remodeling that is unique to the atria.

As in the 2 week group, far fewer gene sets (61 vs. 101) were differentially regulated with 2 day exercise in TNF-KO vs. WT mice. In particular, TNF-KO mice again showed enrichment of NF-κB, toll-like receptor, and interleukin pathways in the sedentary group, further supporting a permissive role for TNF in regulating exercise-induced atrial changes. With regards to individual genes, our analyses showed that fewer atrial genes were differentially regulated between swim WT and KO mice. Of these, the differentially regulated genes related to ECM/mechanosensitive include *Mmp14*, *Tln*1, *Lox*, and *Gsk3*β as well as *Fgf2*, the latter being the only gene upregulated in a TNF-dependent manner exclusively in exercised WT mice. The presence of only one TNF-dependent differentially regulated gene in the WT compared to TNF-KO exercise group is unexpected given the increased filling pressures we observed with swim. However, exercise is an intermittent hemodynamic overload stimulus (Moreira-Goncalves et al., 2015), and the nature and time course of cardiac transcriptomic remodeling in response to mechanical stretch is highly dependent on stretch duration (Rysa et al., 2018). Therefore, as we only looked at atrial transcriptomic remodeling 2 h following the last acute swim bout, and focused our analysis on genes linked to mechanotransduction and ECM remodeling, a larger window is likely necessary to capture the full impact of swim exercise on stretch-induced transcriptomic remodeling and enzyme activity (i.e., TACE/ADAM17, pro-MMP2).

Relative to atria, the number of genes sets in ventricles that were differentially regulated in response to exercise was much smaller. This is not unexpected because ventricles are far less compliant than atria due to differences in ECM as well as wall thickness (La Gerche et al., 2011) in agreement with our previous studies showing that exercise activates p38 in atria but not ventricles (Aschar-Sobbi et al., 2015). Presumably, preferential atrial stretch underlies prominent bi-atrial enlargement in athletes (D’Andrea et al., 2010; D’Ascenzi et al., 2016, 2018) as well as pronounced atrial hypertrophy and fibrosis in our exercised mice. These responses would be expected to normalize atrial wall stress which explains nicely the evolving pattern of time-dependent exercised-induced atrial changes in stretch-dependent signaling pathways in the current study.

Given the link between TNF and mechanical stress, it is tempting to speculate that the degree of TNF elevation with elevated filling pressures and its effects on mechanosensitive signaling cascades may determine the threshold between compensatory (i.e., physiological) or maladaptive transcriptomic activation early in the response to exercise training. This would be consistent with the pleiotropic functions of TNF (Tracey and Cerami, 1994). Indeed, TNF and its downstream factors such as NFκB and p38 can promote protective and pathophysiological responses (Schumacher and Naga Prasad, 2018). Moreover, time-dependent adaptations (i.e., hypertrophy and fibrosis) may serve to blunt or normalize acute elevations in wall stress with exercise, which would explain enrichment in ECM/mechanosensitive genes in sedentary mice over time and compensatory deactivation of stretch-mediated remodeling. This would further promote an early transition to reduced collagen transcription, mimicking fibrotic processes seen in aging wherein collagen expression is also not increased (Podolsky et al., 2020).

### Implications

AF increases strongly with age, CVD and conditions associated with poor cardiovascular health (i.e., diabetes, obesity, and metabolic syndrome). Even though physical activity reduces AF risk (Drca et al., 2014; Malmo et al., 2016), endurance athletes, especially elite veteran athletes, have AF risks rivaling that seen with hypertension and other CVD conditions (Mont et al., 2002; Redpath and Backx, 2015; Goodman et al., 2018). In CVD patients, persistent AF is invariably associated with atrial fibrosis, inflammation and hypertrophy, along with variable electrical changes (Daoud et al., 1996; Xu et al., 2004; Nattel and Harada, 2014). Although historically AF in athletes has often been referred to as “lone AF” (Calvo et al., 2016) because of the absence of CVD, the term is no longer considered appropriate since AF is associated with a multitude of conditions (Wyse et al., 2014). In this regard, animal studies have established, and some human studies suggest, that intense exercise leads to adverse atrial changes resembling those seen in persistent AF patients. In this regard, our studies reveal that exercise induces dynamic transcriptional adaptations involving, in particular, pronounced changes in strain-dependent pathways related to ECM/integrin/focal adhesion. These observations seem particularly relevant since elevated filling pressures and atrial stretch are both prominent features of aging, CVD and exercise. A novel and remarkable finding of our analyses was the link between genes associated with collagen turnover, rather than collagen transcripts, and fibrosis in exercised atria, a pattern that mirrors aging-related fibrosis (Podolsky et al., 2020). This is especially interesting because the strongest predictor of AF is age (Staerk et al., 2018). Moreover, our findings revealed TNF plays a permissive rather than primary role in exercise-mediated atrial structural and transcriptomic remodeling.

TNF involvement in exercise-induced structural and transcriptional adaptations are of particular interest because TNF has been implicated in the pathogenesis of AF (Ren et al., 2015) and persistent AF is associated with elevated atrial TNF levels and inflammatory infiltrates (Li et al., 2010; Guo et al., 2012). Collectively, the many common atrial features between persistent AF patients and exercised mice suggests to us that AF and adverse remodeling seen with intense exercise and CVD share common mechanisms. Thus, while our findings of an arrhythmogenic substrate requires confirmation in athletes presenting with AF, the genetic changes seen in our studies may have broader implications for the general AF population. By comparison, ventricular responses to exercise were relatively muted, although the differentially regulated gene sets were similar to those in the atria, consistent with clinical and epidemiological evidence for exercise-induced arrhythmogenic remodeling being chamber-specific (Guasch et al., 2018).

### Limitations

Obviously the use of whole tissue samples prevents us from determining the individual contributions of cardiomyocytes, endothelial cells, and fibroblasts to the transcriptomic remodeling induced by exercise which would be highly desirable since TNF is a mechanosensitive cytokine expressed in cardiomyocytes (Kapadia et al., 1997; Sun et al., 2007), fibroblasts (Yokoyama et al., 1999), and endothelial cells (Yin et al., 2017).

Although the studies presented here were limited to male mice, we have plan to examine the effects of exercise on female mice. It is worth noting that even though female athletes remain underrepresented generally in previous studies examining exercise-induced AF (Andersen et al., 2013), several studies have found a reduced incidence of AF in females (Mohanty et al., 2016) making our future studies potentially highly relevant.

Our studies were limited to only three time points leaving many uncertainties regarding the evolving effects of intense exercise. Nevertheless, our results showed time-dependent adaptations to exercise with generally comparable responses at the 2 and 6 week time points, suggestive of compensatory adaptations in fibrotic, hypertrophic and inflammatory pathways. In this regard, we did not extend our swim training beyond 6 weeks given the clear evidence of adverse atrial remodeling and increased AF vulnerability at this time point. However, given the presence of inflammatory infiltrates and enrichment of TNF-mediated inflammatory signaling pathways at 6 weeks of exercise, it is conceivable that the arrhythmogenic substrate and the degree of AF vulnerability may be more pronounced if we were to extend our exercise protocol. We cannot rule out increased collagen synthesis or mechanisms involved in post-translational modifications/deposition (i.e., SPARC/OPN, LOXs) or degradation (i.e., MMPs) as contributing to exercise-induced atrial remodeling, which might not have been fully captured at 2 day, 2 or 6 week exercise.

While we used microarray analyses for 6 week data and RNAseq for the other time points to assess exercise-induced transcriptional changes, our analyses focused on RNAseq data at the earlier time points. In this regard, it was reassuring to find that the 6 weeks microarray results align well with the 2 week RNAseq results, thus robustly supporting the compensatory nature of the atrial responses to exercise at the later time points.

## Conclusion

Our results demonstrate clear exercise-induced TNF-dependent differential activation (enrichment) of pathways associated with mechanosensitive ECM remodeling, which are both time-dependent and differ between atria and ventricles in a manner consistent with preferential stretch of atria in response to exercise-induced elevations in venous pressure. Our findings provide insight into the chamber-specific roles of TNF and mechanical strain in cardiac changes induced by exercise, which supports the general conclusion that exercise-induced adverse atrial remodeling and AF vulnerability is linked to elevated filling pressures, consistent with AF associated with aging and poor cardiovascular health. The common atrial features between persistent AF patients and exercised mice suggests that AF and adverse remodeling seen with intense exercise may share common mechanisms, which may have broader implications for the general AF population.

## Data Availability Statement

The data generated in this study can be found in the BioProject database (accession: PRJNA663094).

## Ethics Statement

The animal study was reviewed and approved by the Division of Comparative Medicine at the University of Toronto and York University Animal Care Committee (ACC).

## Author Contributions

Experiments were performed at York University Department of Biology, the University of Toronto Department of Physiology, and the University of Alberta Department of Physiology. YO, SY, RL, and PB were responsible for the conception and design of the work, the acquisition, analysis, and interpretation for the work, drafting the work, and revising it critically for important intellectual content. XL, SJ, FI, XG, RD, and D-KK were involved in acquisition, analysis, and interpretation of data for the work. All authors approved the final version of this manuscript and agreed to be accountable for all aspects of the work.

## Conflict of Interest

The authors declare that the research was conducted in the absence of any commercial or financial relationships that could be construed as a potential conflict of interest.
