# Supplementary material for: Transcriptomic Bioinformatic Analyses of Atria Uncover Involvement of Pathways Related to Strain and Post-translational Modification of Collagen in Increased Atrial Fibrillation Vulnerability in Intensely Exercised Mice
Source: Front Physiol. 2020 Dec 23;11:605671. doi: 10.3389/fphys.2020.605671 (PMC7793719; doi:10.3389/fphys.2020.605671)
Supplement: Supplementary file 1 [file Data_Sheet_1.docx]

Supplementary Material

# Supplementary Figures


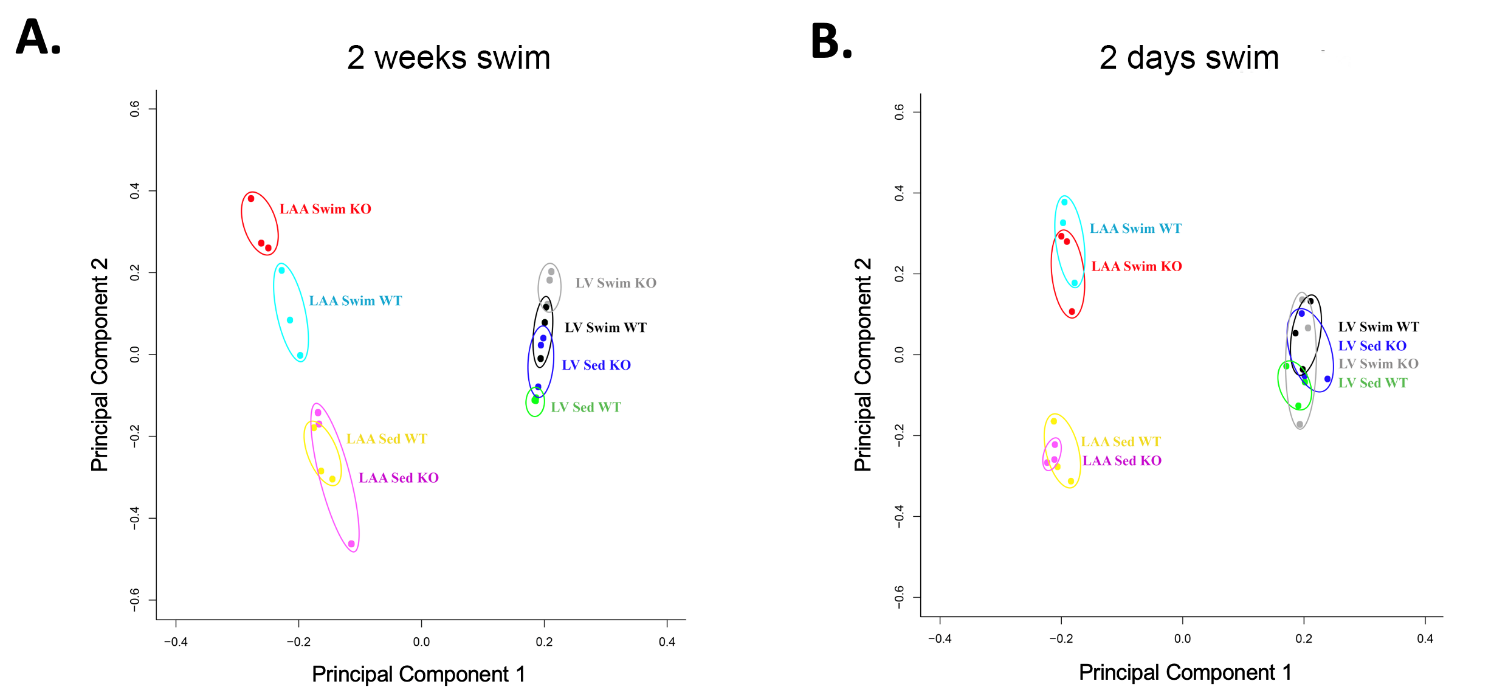


**Supplementary Figure 1. Principal component analysis (PCA) of all samples and its technical replicates. (A)** PCA for 2-week swim and sedentary left atrial appendage (LAA) and left ventricular (LV) samples for wild-type (WT) and tumor necrosis factor (TNF) knockout (KO) mice. **(B)** PCA for 2-day tissue samples. PCA plots show clear clustering of separate sample groups using the greatest variation (principal component 1) that lies between different sample groups. For both 2-week and 2-day samples, technical replicates (2 replicates per sample) show the least amount of variation, and the greatest amount of variation lies between the LAA and LV samples.


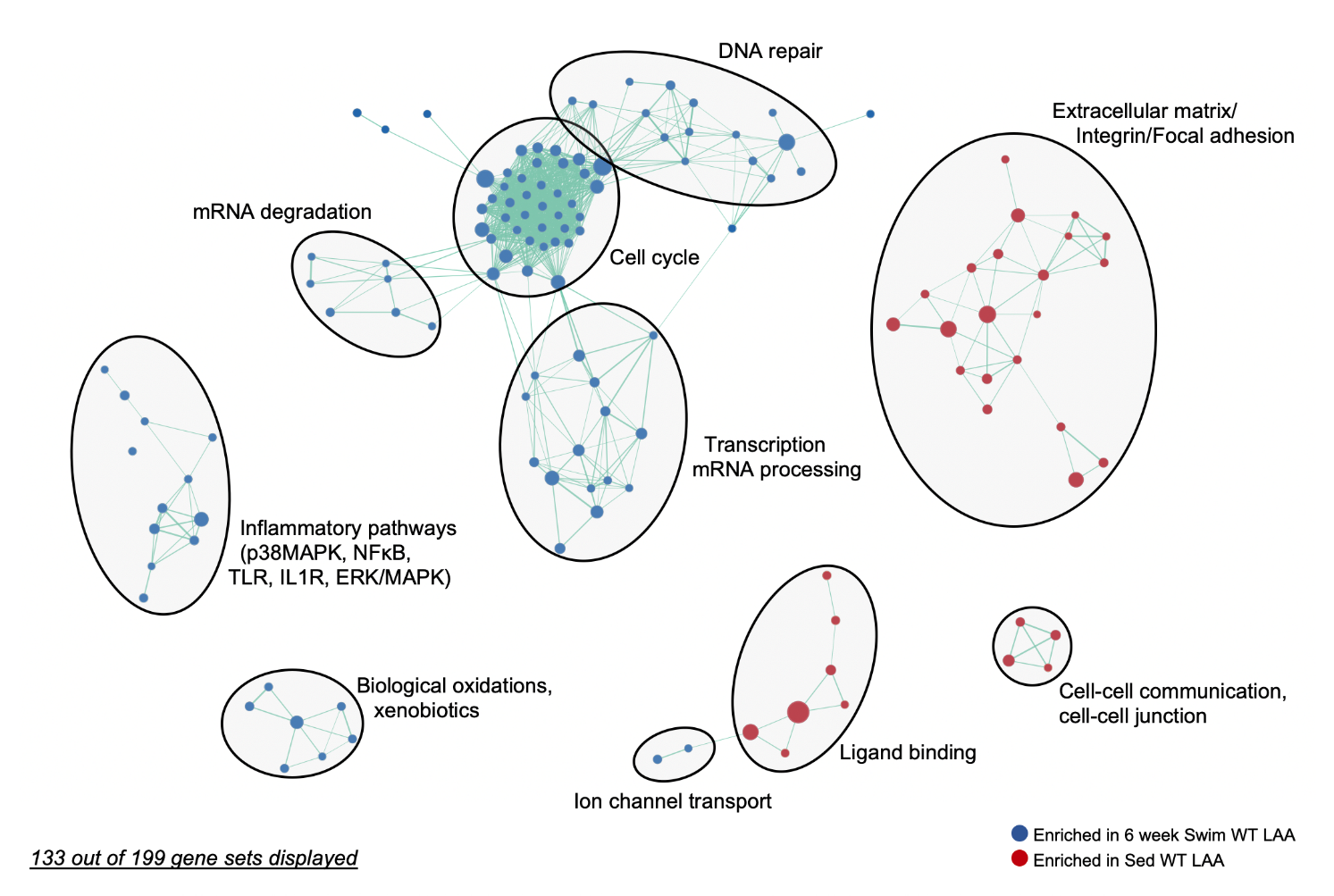


**Supplementary Figure 2. Intense 6-week swim exercise-induced atrial transcriptomic remodeling.** Gene set enrichment analysis (GSEA) and enrichment mapping of microarray data revealed clusters of differentially regulated pathways in the left atrial appendage (LAA) between wild-type (WT) 6-week swim and sedentary mice. Blue dots indicated gene set enrichment in 6-week swim mice, while red dots indicated enrichment in sedentary mice. Only gene sets that form clusters are shown for clarity (133 out of 199 gene sets), with connecting lines indicating gene set overlap. Nominal P-value <0.05, false discovery rate (FDR) <0.20.


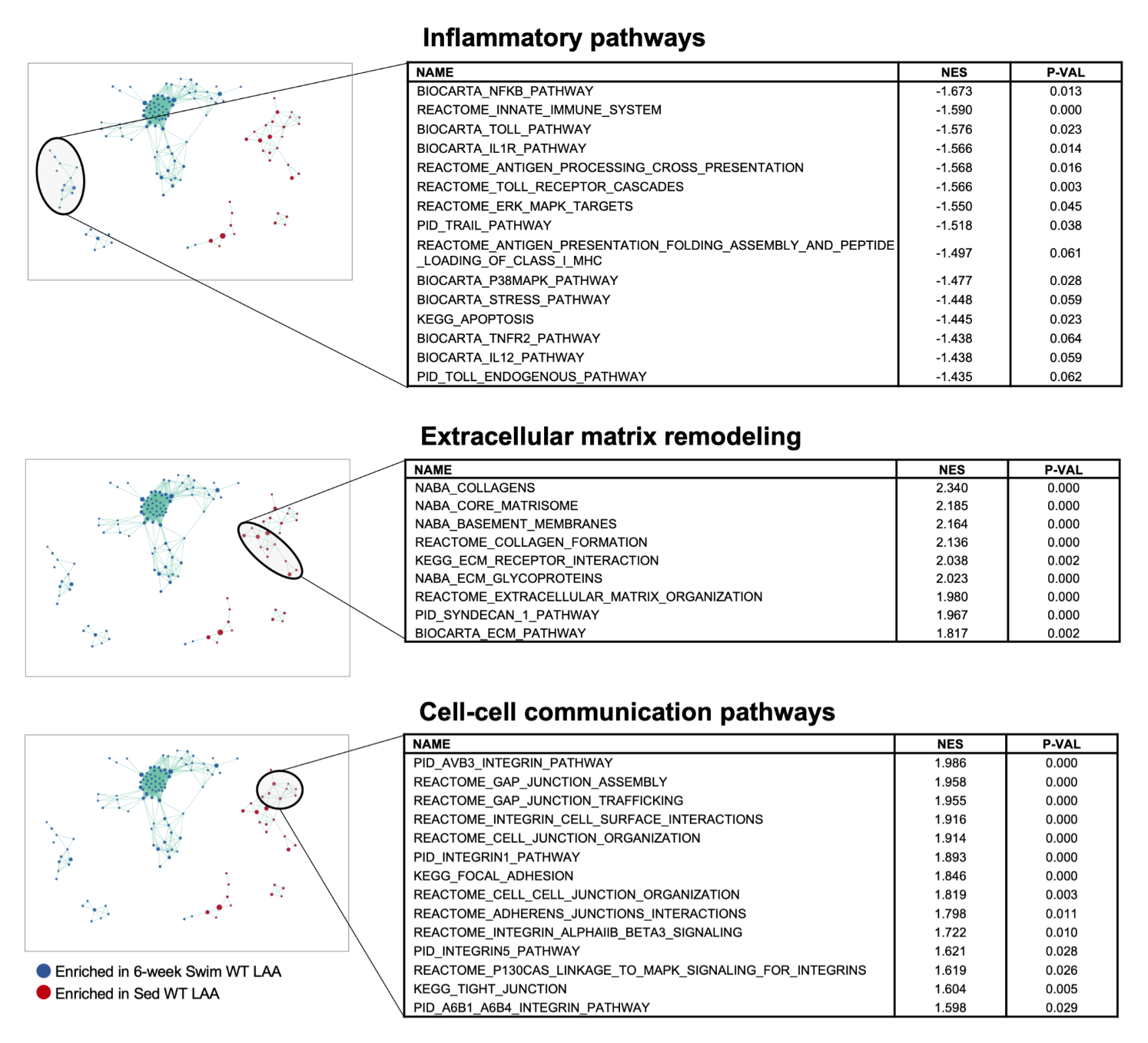


**Supplementary Figure 3. Summary of differentially regulated gene sets related to inflammatory pathways, extracellular matrix remodeling, and cell-cell communication pathways with 6-week swim exercise.** Exercise induces enrichment of inflammation related pathways (NF-κB, p38 MAPK, interleukin-1(IL1R) and toll signaling pathways. Gene sets related to mechanosensitive pathways (i.e. focal adhesion kinases (FAK), integrins, cell-cell communication) and extracellular matrix (ECM) remodeling (i.e. collagen formation, degradation, biosynthesis, assembly, cross-linking and matrisome enzymes) are enriched in the sedentary atria.


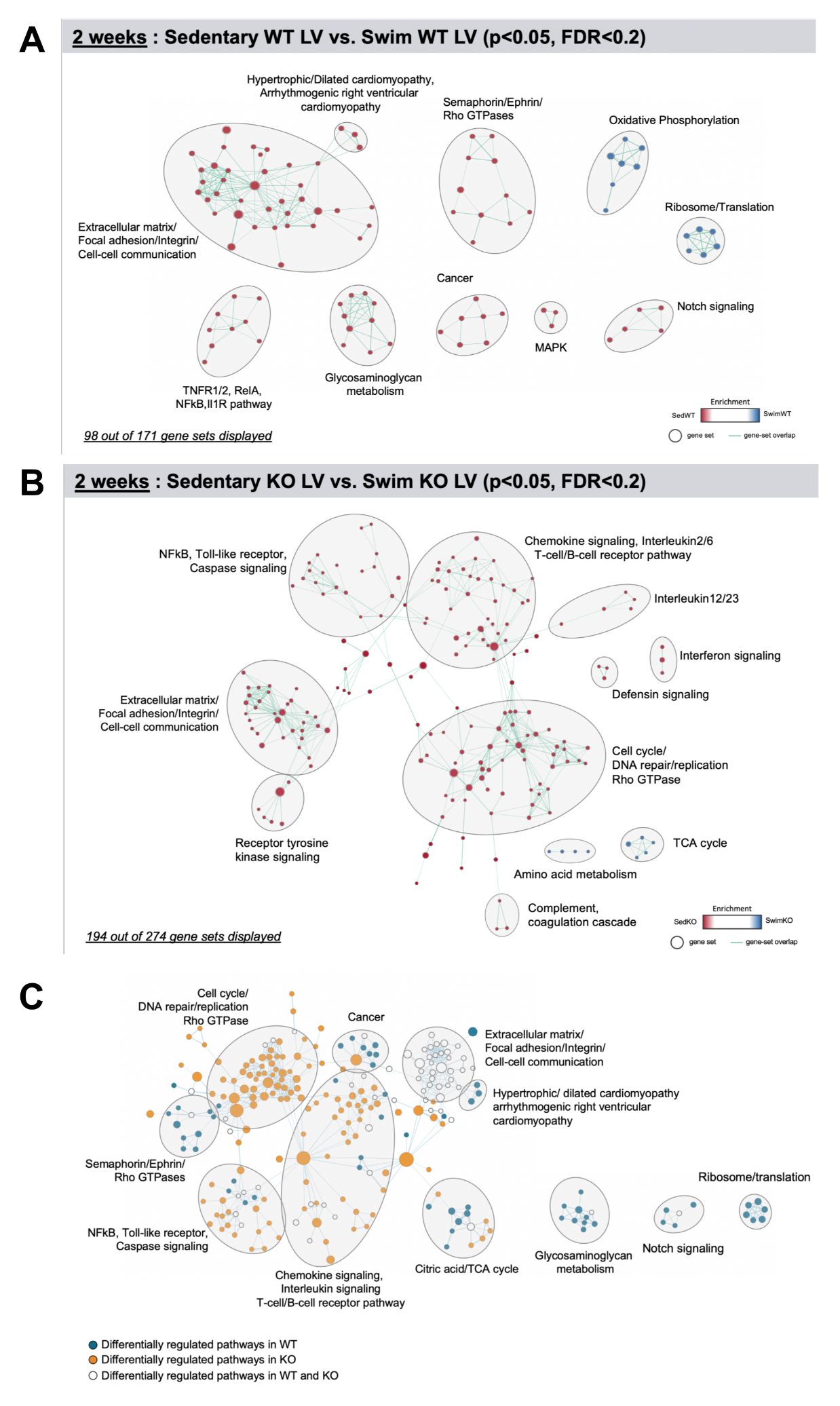


**Supplementary Figure 4. Differentially regulated pathways in the left ventricle (LV) of 2-week swim exercised wild-type (WT) and tumor necrosis factor (TNF) knockout (KO) mice. (A)** Gene set enrichment analysis (GSEA) and enrichment mapping showing clusters of differentially regulated pathways in the left ventricle (LV) between WT 2-week swim (blue dots) and sedentary (red dots) mice. **(B)** Enrichment map showing clusters of differentially regulated pathways in the LV of TNF KO 2-week swim (blue dots) and sedentary (red dots) mice. **(C)** Enrichment map of the difference of the difference analysis revealing clusters of exercise-induced differentially regulated pathways in WT (blue dots) versus TNF KO (orange dots) mice. Only gene sets that form clusters of ≥5 gene sets are shown for clarity, with connecting lines indicating gene set overlap. Nominal P-value <0.05, false discovery rate (FDR)<0.20.


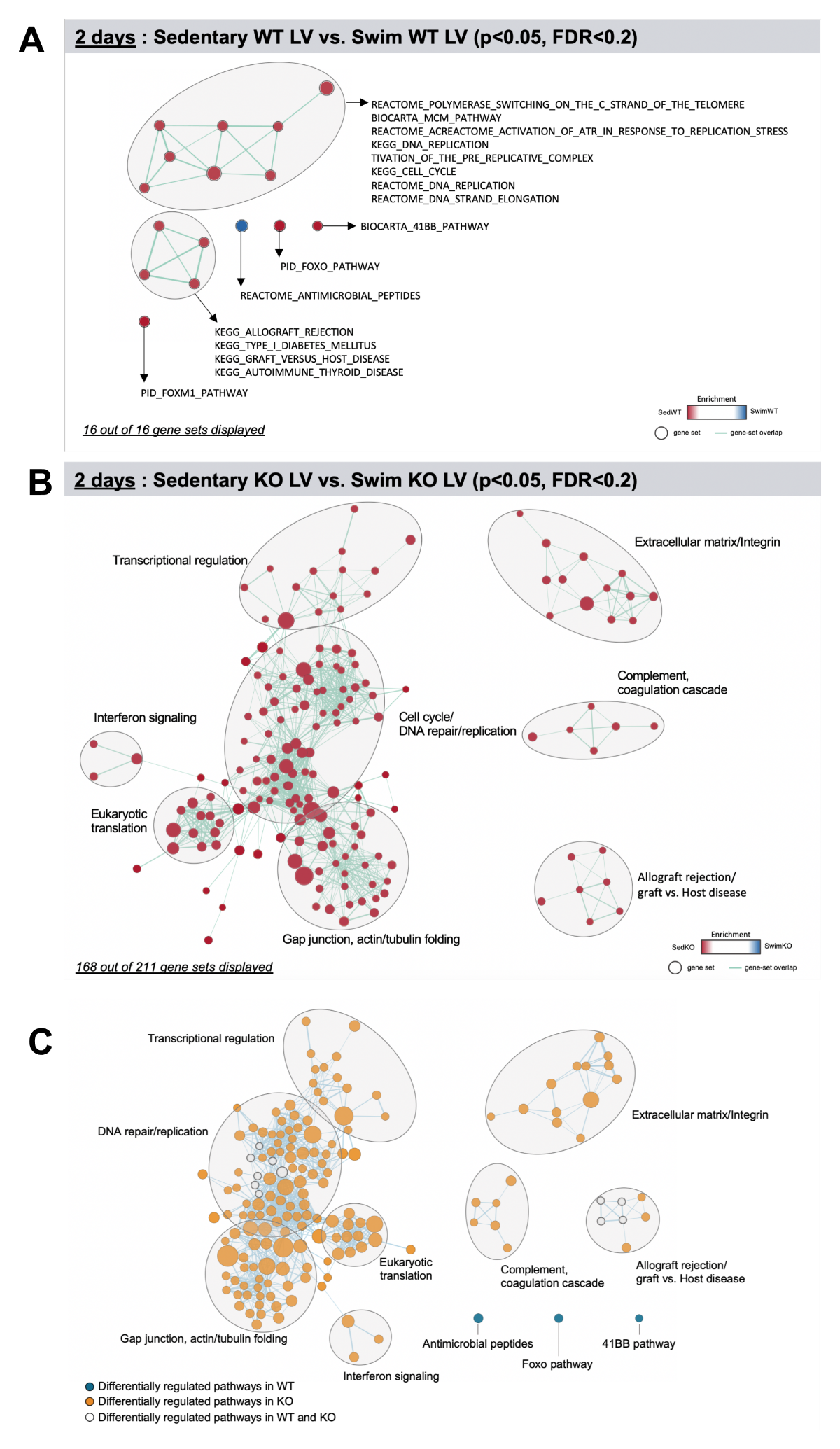


**Supplementary Figure 5.** **Differentially regulated pathways in the left ventricle (LV) of 2-day swim exercised wild-type (WT) and tumor necrosis factor (TNF) knockout (KO) mice. (A)** Gene set enrichment analysis (GSEA) and enrichment mapping showing clusters of differentially regulated pathways in the left ventricle (LV) between WT 2-day swim (blue dots) and sedentary (red dots) mice. **(B)** Enrichment map showing clusters of differentially regulated pathways in the LV of TNF KO 4-session swim (blue dots) and sedentary (red dots) mice. **(C)** Enrichment map of the difference of the difference analysis revealing clusters of exercise-induced differentially regulated pathways in WT (blue dots) versus TNF KO (orange dots) mice. Only gene sets that form clusters are shown for clarity, with connecting lines indicating gene set overlap. Nominal P-value <0.05, false discovery rate (FDR)<0.20.


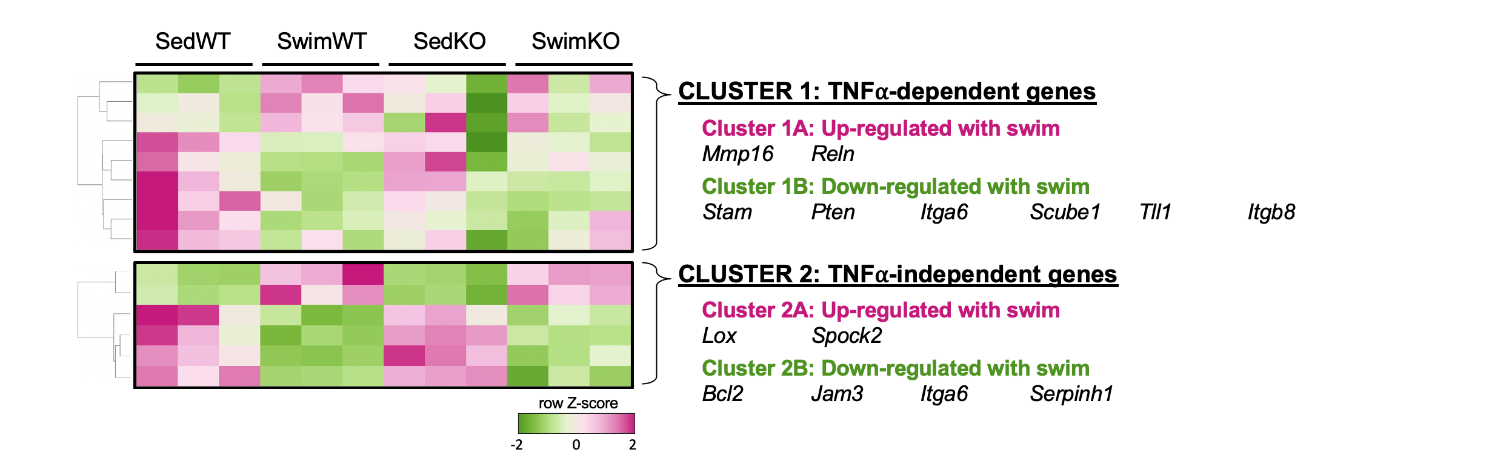


**Supplementary Figure 6. Heat map and clustering analysis of the tumor necrosis factor (TNF)-dependence of extracellular matrix (ECM)-, integrin-, and cell-cell communication-associated genes in the left ventricle (LV) of 2-day swim exercised mice.** Heat map and cluster analysis of individual genes belonging to ECM-, integrin-, and/or cell-cell communication-associated gene sets that are up- or down-regulated with swim exercise in TNF-dependent (cluster 1) or TNF-independent (cluster 2). The order of genes (top to bottom) corresponds to the gene list (left to right). TNF-dependent (cluster 1) is: SedWT vs SwimWT (P<0.05) and SedKO vs Swim KO (not P<0.05). TNF-independent (cluster 2) is: SedWT vs SwimWT (P<0.05) and SedKO vs SwimKO (P<0.05).


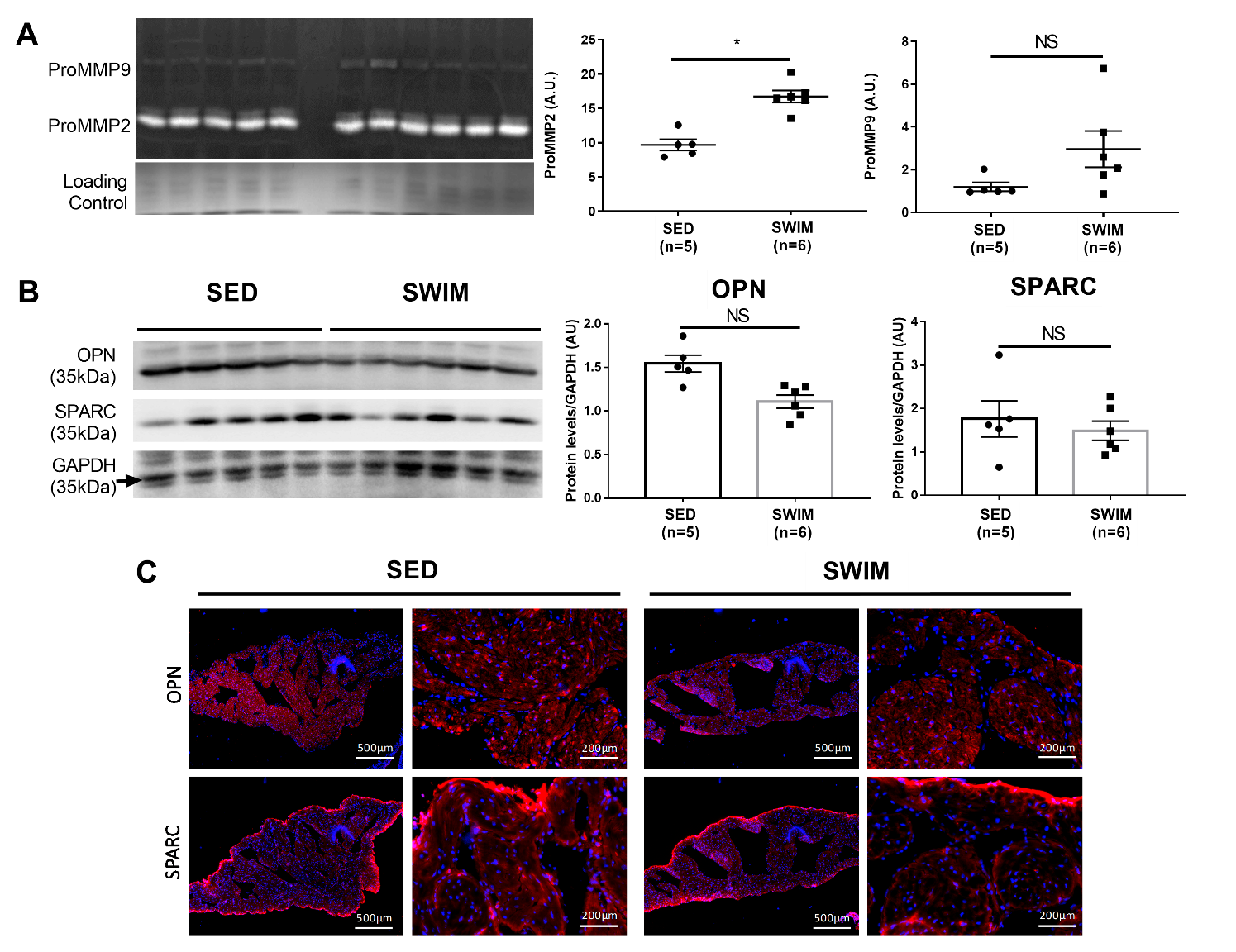


**Supplementary Figure 7. MMP2 and MMP9 activity levels and matricellular protein and immunohistochemical levels in 2-day sedentary and swim mice. (A)** Representative in vitro gelatin zymography and averaged band intensities for pro-MMP2 and pro-MMP9 in sedentary and swim left atrial appendages. **(B)** Representative Western blots for osteopontin (OPN) and secreted protein acidic and rich in cysteine (SPARC). Black arrow indicates the GAPDH band. Quantification of the protein levels for OPN and SPARC for sedentary (n=5) and swim (n=6) left atrial appendages. GAPDH served as the loading control. (**C)** Representative immunofluorescent images for OPN and SPARC straining from left atrial appendages of sedentary and swim mice at 4x and 20x magnification. Data represented mean ± SEM. *P<0.05 compared to sedentary control. AU indicates arbitrary units.
